# An AI-Assisted Workflow for Rapid Prioritization of FDA-Approved Drugs as HDAC3 Inhibitor Candidates for Drug Repurposing

**DOI:** 10.3390/biology15181570

**Published:** 2026-09-08

**Authors:** Aoi Kunimoto, Valentina L. Kouznetsova, Santosh Kesari, Igor F. Tsigelny

**Affiliations:** 1 California Institute of Technology, Pasadena, CA 91125, USA; akunimot@caltech.edu; 2 Mentor Assistance Program, University of California San Diego, La Jolla, CA 92093, USA; 3 San Diego Supercomputer Center, University of California San Diego, La Jolla, CA 92093, USA; 4 BiAna Institute, La Jolla, CA 92038, USA; 5 Lundquist Institute, Torrance, CA 90502, USA; 6 Department of Neurosciences, University of California San Diego, La Jolla, CA 92093, USA

**Keywords:** epigenetic regulation, histone deacetylase 3, cancer, machine learning

## Abstract

Epigenetic regulation through histone acetylation plays a critical role in gene expression and cancer progression. Because of its pivotal role in chromatin remodeling, Histone deacetylase 3 (HDAC3) has become a promising therapeutic target. In this study, an artificial intelligence (AI)-driven strategy was utilized to prioritize potential HDAC3 inhibitors among FDA-approved compounds to accelerate drug repurposing for cancer therapy. Existing HDAC3 inhibitors were identified in the BindingDB and were used to develop a machine learning (ML) model trained on the most potent inhibitors to identify molecular descriptor patterns associated with HDAC3 inhibition. The ML workflow then screened 1615 FDA-approved compounds, yielding 120 candidates with predicted HDAC3 inhibitory activity. Among these, known HDAC inhibitors, including romidepsin, vorinostat, and panobinostat, were selected, suggesting that the workflow can recover known HDAC inhibitors during virtual screening. Interestingly, tyrosine kinase inhibitors such as imatinib and osimertinib were also identified, indicating potential structural overlap between kinase- and HDAC3-binding pharmacophores. The analysis of the predicted docking scores also supported the prioritization results since the top 10 compounds had more negative predicted docking scores than the bottom 10 (*p* = 0.0074). This shows that the suggested workflow is useful for prioritizing FDA-approved compounds as potential HDAC3 inhibitors for further study.

## Simple Summary

Cancer development is often driven by abnormal changes in how cells control genes. A specific protein called histone deacetylase 3 plays a major role in these changes, making it an attractive target for new treatments. Finding new therapies is highly time-consuming, so repurposing safe, existing drugs is a vital strategy. Here, our objective was to use artificial intelligence to quickly screen 1615 compounds already approved by the Food and Drug Administration to see if they could block histone deacetylase 3. By training a computer program to recognize chemical patterns, we screened these approved drugs and identified 120 potential candidates. We then used molecular docking, a computational method that simulates how well a compound fits into a protein, like a key in a lock, to evaluate them. As a control, the system successfully recovered drugs already known to block the target protein, indicating that the computational approach works. In addition, the screening captured many other drugs, prominently including tyrosine kinase inhibitors, another major class of modern anti-cancer drugs. This suggests these existing drugs may also have additional blocking activity. For society, this strategy could significantly reduce the time and resources required to discover effective cancer therapies, thereby accelerating the development of new treatments for patients.

## 1. Introduction

Cancer remains one of the leading causes of mortality worldwide and continues to be an incurable disease despite significant advances in detection and treatment. Conventional therapies such as surgery, chemotherapy, and radiation have improved outcomes for certain cancer types. However, many malignancies remain resistant to treatment or prone to relapse [1,2]. In recent decades, the application of genetic and molecular insights has opened new therapeutic avenues, offering targeted approaches that address the underlying mechanisms of tumorigenesis [3]. Among these, the study of genetic mutations and epigenetic dysregulation has emerged as a critical component in understanding and treating cancer.

Cancer develops via complex mechanisms, including the accumulation of genetic mutations which turn on oncogenes or switch off tumor suppressor genes, thereby disrupting the normal control of the cell cycle and leading to uncontrolled proliferation. Epigenetic changes also play a major role in the development and progression of tumors. Unlike mutations, epigenetic modifications do not change the DNA sequence; rather, they influence gene expression via mechanisms such as DNA methylation, histone modifications, and non-coding RNAs [4,5,6,7]. The fact that epigenetic alterations are reversible is what makes them so suitable as therapeutic targets.

Among these mechanisms, histone modifications, especially acetylation and deacetylation, are key regulators of chromatin structure and gene expression. Aberrant activity of histone-modifying enzymes such as HDACs has been implicated in several cancer types, contributing to the silencing of tumor suppressor genes and the activation of oncogenic pathways.

### 1.1. Histone Deacetylases in Transcriptional Regulation

The acetylation of histones, a process carried out by histone acetyltransferases (HATs), causes the chromatin structure to become more relaxed and thus promotes transcription. On the other hand, histone deacetylases (HDACs) remove acetyl groups from the lysine residues on the histone tails, resulting in chromatin condensation and the silencing of genes [8]. These HDACs are enzymes that have been conserved throughout evolution and are divided into four different classes according to their sequence homology and their reliance on particular cofactors. The HDACs in Classes I, II, and IV are zinc-dependent, while those in Class III, which are called sirtuins, need NAD^+^ in order to be active [9].

Each HDAC class possesses unique structural features that confer diverse biological functions. For instance, Class I HDACs, particularly HDAC1, HDAC2, and HDAC3, are ubiquitously expressed and primarily localized to the nucleus, where they regulate critical pathways involved in cell cycle progression, DNA repair, and apoptosis [10].

### 1.2. HDAC Dysregulation and Oncogenesis

Aberrant expression or activity of HDACs has been widely implicated in oncogenesis. The Cancer Genome Atlas (TCGA) and other large-scale epigenomic studies have reported dysregulated HDAC expression across a wide spectrum of malignancies, including breast, lung, liver, colon, stomach, prostate, bladder, and ovarian cancers, as well as hematologic malignancies such as acute myeloid leukemia (AML), acute lymphoblastic leukemia (ALL), chronic lymphocytic leukemia (CLL), diffuse large B-cell lymphoma (DLBCL), and Hodgkin lymphoma [11,12]. These alterations frequently correlate with poor prognosis, therapy resistance, and aggressive clinical phenotypes.

HDACs play a mechanistic role in the development and progression of cancer by suppressing tumor suppressor genes, disrupting DNA repair pathways, and aiding in the maintenance of cancer stem cells. Their functional versatility is mainly due to their capacity to interact with a wide variety of transcriptional regulators and core-repressor complexes, which makes them key modulators of both oncogenic and tumor-suppressive networks [10].

### 1.3. HDAC3: A Central Player in Tumorigenesis

HDAC3, which belongs to the Class I HDACs, has a crucial role in cancer biology. It acts as the catalytic component of the nuclear receptor corepressor (NCoR) and the silencing mediator of retinoic acid and thyroid hormone receptor (SMRT) complexes, complexes that are necessary for its enzymatic activity. By means of these complexes, HDAC3 brings about transcriptional repression programs that are important for cellular proliferation and survival [13,14,15]. Abnormal regulation of HDAC3 has been recorded in a variety of solid and blood cancers. In the case of breast cancer, HDAC3 is markedly overexpressed, especially in aggressive subtypes and in cases resistant to aromatase inhibitors, and its activity is linked to tumor progression and resistance to treatment [16,17]. In colorectal cancer, overexpression of HDAC3 leads to changes in epigenetic regulation, disruption of Wnt signaling, and reduced p21 expression, thereby promoting uncontrolled proliferation [18,19,20,21]. In prostate cancer, HDAC3 has been involved in tumorigenesis, and its expression is affected by dietary compounds that act within tumor-suppressive pathways [22,23]. Similarly, in hepatocellular carcinoma, HDAC3 promotes cancer cell proliferation through the STAT3 signaling pathway [24]. In acute myeloid leukemia (AML), HDAC3 has been linked to chemoresistance via modulation of DNA damage response pathways [25,26]. In diffuse large B-cell lymphoma (DLBCL), HDAC3 expression differs between malignant and normal tissues, with emerging evidence indicating its prognostic relevance [27,28]. Notably, although not always specific to HDAC3, broad-spectrum HDAC inhibitors such as valproic acid have demonstrated cytotoxic effects in pediatric brain tumors, including medulloblastoma, supporting the therapeutic potential of targeting HDACs in central nervous system malignancies [29,30,31].

Functionally, HDAC3 promotes oncogenesis by silencing tumor suppressor genes, disrupting DNA damage response pathways, and fostering chromosomal instability [32,33,34]. Silencing or inhibition of HDAC3 in preclinical models has been shown to induce apoptosis, inhibit proliferation, and enhance the sensitivity of tumor cells to chemotherapeutic agents [35]. These insights underscore the value of HDAC3 as a therapeutic target and justify its growing prominence in epigenetic drug discovery efforts.

### 1.4. Therapeutic Targeting of HDACs and HDAC3

The clinical relevance of HDAC inhibition has been validated by the FDA approval of several histone deacetylase inhibitors (HDACis), including romidepsin, vorinostat, and panobinostat, primarily for the treatment of hematologic malignancies [36]. These agents exert anti-cancer effects by reactivating silenced genes, inducing cell cycle arrest, promoting apoptosis, and enhancing tumor immunogenicity [37].

However, the broad activity spectrum of pan-HDAC inhibitors often leads to dose-limiting toxicities due to non-specific inhibition of multiple HDAC isoforms. This has spurred efforts to develop isoform-selective inhibitors with enhanced efficacy and reduced toxicity profiles. In particular, structure-based drug design has made significant strides in targeting HDAC3 specifically. The structural elucidation of HDAC3 in complex with its corepressors has enabled the development of HDAC3-selective inhibitors, such as RGFP966 and BRD3308, which demonstrate potent anti-tumor effects with improved safety margins in both in vitro and in vivo models [38,39,40,41,42,43].

### 1.5. Leveraging Artificial Intelligence in HDAC3 Inhibitor Discovery

Given HDAC3’s well-documented role in cancer progression and therapy resistance, it represents a compelling target for drug development. Recent advances in artificial intelligence (AI) and machine learning (ML) have revolutionized drug discovery pipelines, enabling high-throughput virtual screening, target prediction, and compound optimization [44]. In our previous work, we demonstrated the effectiveness of AI-driven virtual screening in identifying novel inhibitors against HIV-1 protease, repurposing FDA-approved compounds to accelerate antiviral drug discovery [45].

Building upon our previous work, we developed an AI-assisted workflow to prioritize potential HDAC3 inhibitors within the FDA-approved drug space for drug repurposing. Experimentally validated HDAC3 inhibitors retrieved from the first public molecular recognition database, BindingDB [46], were used to identify molecular descriptor patterns associated with HDAC3 inhibition. The resulting ML workflow was then applied to screen 1615 FDA-approved compounds, followed by structure-based computational characterization. This workflow prioritized multiple promising FDA-approved compounds, including known HDAC inhibitors as well as structurally diverse candidates, demonstrating its utility as a practical strategy for HDAC3-targeted drug repurposing [47,48].

## 2. Results and Discussion

Our study successfully established an AI-assisted workflow for prioritizing FDA-approved compounds as candidate Histone Deacetylase 3 (HDAC3) inhibitors for cancer drug repurposing. This workflow prioritized 120 FDA-approved compounds with predicted HDAC3 inhibitory activity. Known HDAC inhibitors, including romidepsin, vorinostat, and panobinostat, were recovered among the prioritized compounds, providing a useful internal consistency check that the descriptor- and fingerprint-based model retained molecular patterns associated with the positive group.

### 2.1. Classifier Performance in WEKA Screening for HDAC3 Inhibitor Prioritization

A machine learning workflow centered on the Random Forest classifier was used to prioritize 120 FDA-approved candidate HDAC3 inhibitors. The model was developed using a balanced dataset comprising a positive group of known HDAC3 inhibitors curated from BindingDB and a reference comparison group randomly selected from FDA-approved compounds in the ZINC20 database to represent the FDA-approved chemical space for drug repurposing. After excluding prodrugs from both groups, the final training dataset consisted of 291 compounds (148 positive compounds and 143 compounds in the reference comparison group).

Further details of the dataset preparation are provided in Section 4.1.

**Figure 1:**
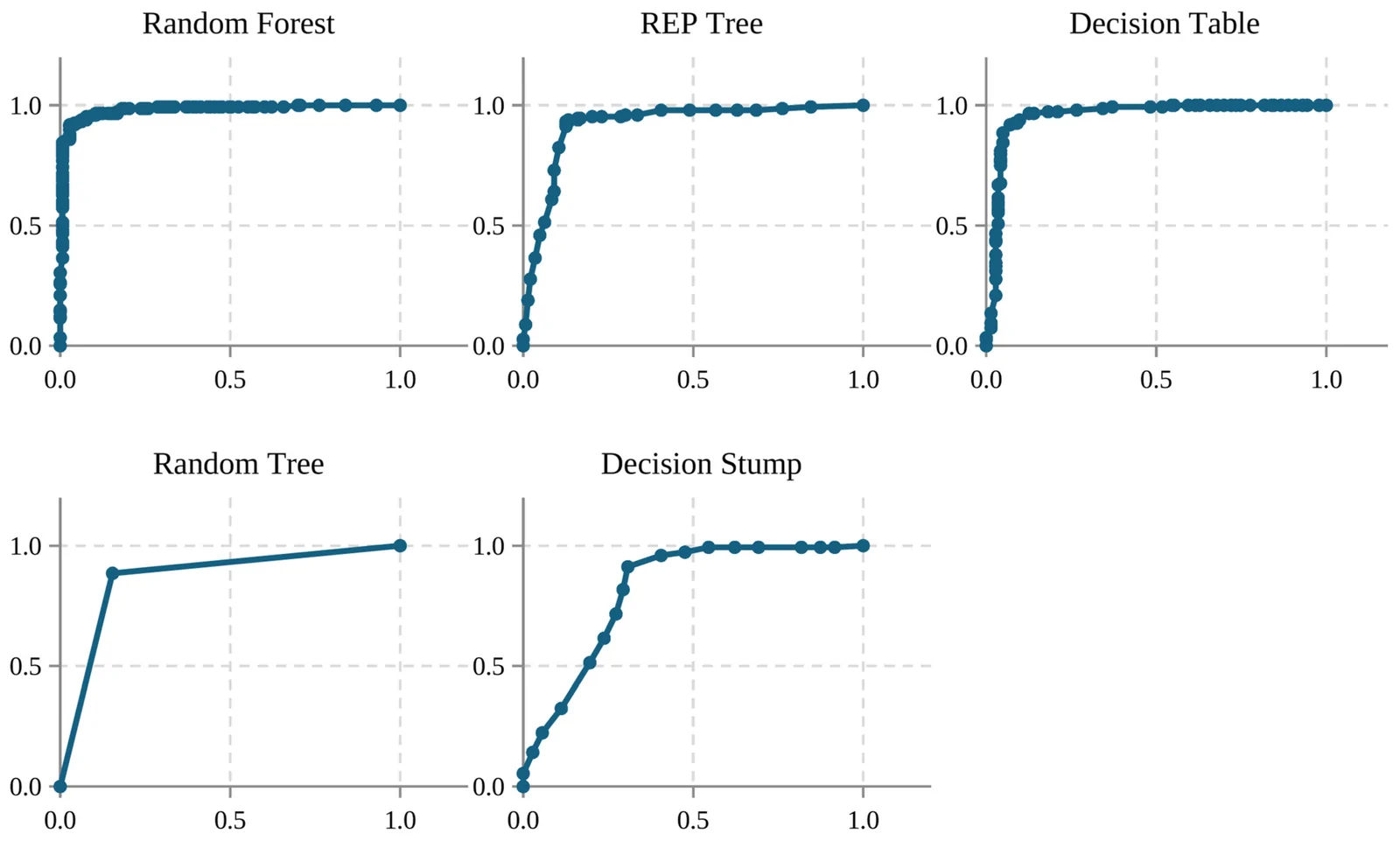
ROC Curve Analysis of Machine Learning Classifiers. Individual receiver operating characteristic (ROC) curves for each classifier. The ROC curves visually represent the classifier’s ability to distinguish between the positive group and the reference comparison group by plotting the True Positive Rate (*y*-axis) against the False Positive Rate (*x*-axis) at various threshold settings. The closer a curve is to the top-left corner, the better the model’s performance. All ROC curves were generated from stratified 10-fold cross-validation performed in WEKA.

**Figure 2:**
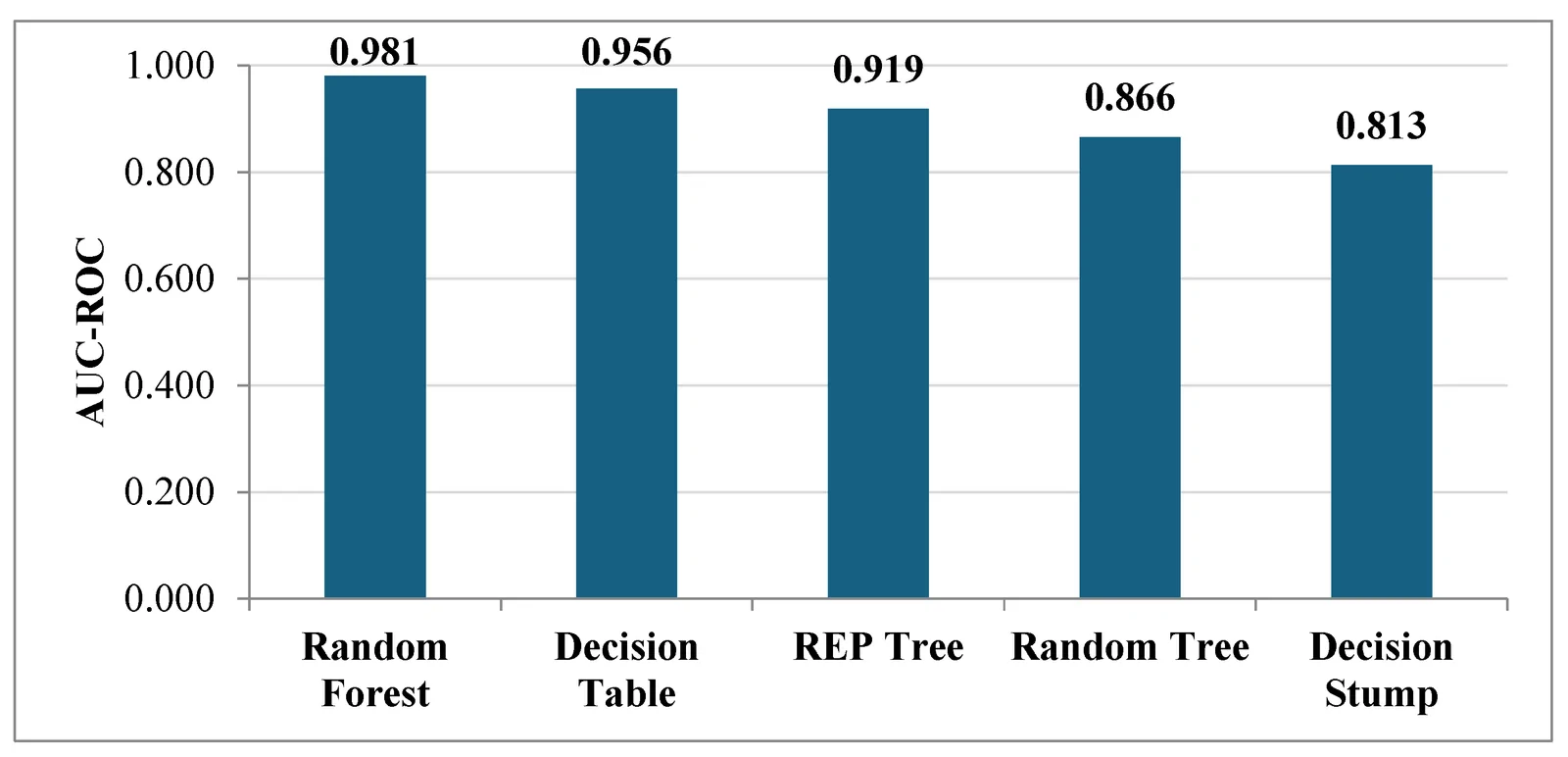
Comparison of AUC-ROC Scores. This bar chart provides a clear visual comparison of the AUC-ROC scores of the five classifiers. The graph reinforces the ranking presented in Figure 1, with taller bars indicating a higher AUC-ROC score and thus a better overall predictive performance for prioritizing candidate HDAC3 inhibitors. AUC-ROC values were calculated using stratified 10-fold cross-validation performed in WEKA.

For each compound in this dataset, 4179 PaDEL-derived molecular descriptor and fingerprint features were used as input variables for machine learning. These features represented complementary physicochemical, topological, and molecular substructure information. To select the most effective classifier, we evaluated five different WEKA classifiers: Random Forest, REPTree, Decision Table, Random Tree, and Decision Stump. As shown in Table 1, the Random Forest model achieved the highest cross-validated performance among the five classifiers evaluated, with an AUC-ROC of 0.981 and an accuracy of 93.1%. The ROC curves are shown in Figure 1, and the AUC-ROC values of the evaluated classifiers are compared in Figure 2.

To improve model interpretability, feature importance was evaluated based on the average impurity decrease implemented in the WEKA Random Forest classifier. The analysis identified multiple molecular features contributing to model prediction. The 30 highest-ranking features are summarized in Supplementary Table S1. Analysis of the highest-ranked features identified descriptors related to molecular topology and connectivity (e.g., PJI2 and MPC04), topological charge distribution (e.g., JGT and GGI2), and polarizability-weighted autocorrelation (e.g., ATS4p). These features suggest that molecular topology, charge distribution, and polarizability contribute to the model’s classification and are broadly consistent with the physicochemical constraints of the HDAC3 catalytic pocket.

Finally, the proposed workflow was applied to an expanded set of 1615 FDA-approved compounds, prioritizing 120 candidate HDAC3 inhibitors for further investigation.

### 2.2. Therapeutic Distribution of Identified Candidate HDAC3 Inhibitors

**Figure 3:**
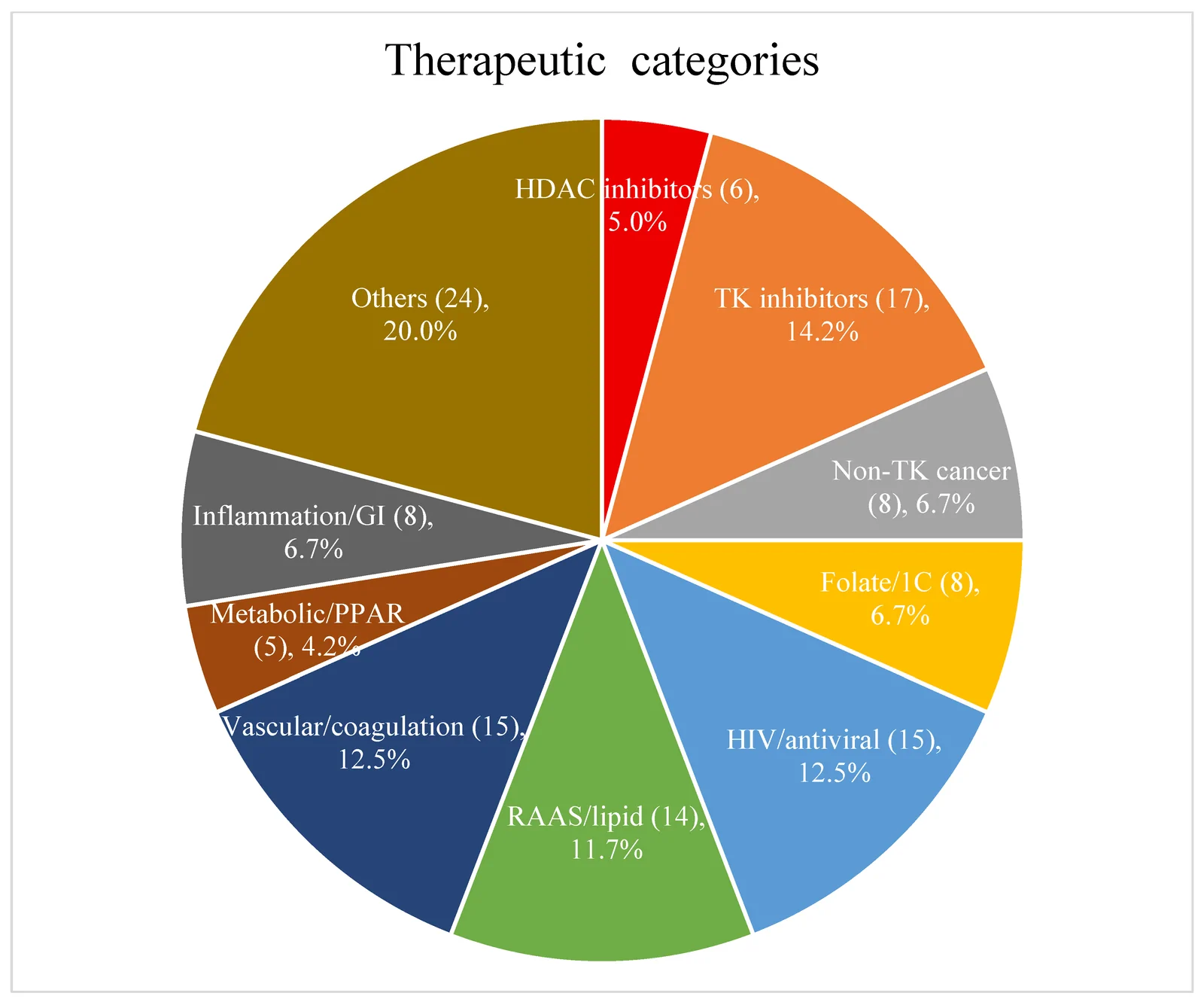
Therapeutic Category Distribution of Potential HDAC3 Inhibitors Identified by AI Screening. Pie chart illustrating the percentage distribution of the 120 FDA-approved compounds predicted as potential HDAC3 inhibitors by our AI-assisted workflow across various therapeutic categories. Each segment represents a distinct drug category, highlighting the chemical and pharmacological diversity of the identified hits. Abbreviations: TK, tyrosine kinase; RAAS, renin–angiotensin–aldosterone system; PPAR, peroxisome proliferator-activated receptor; GI, gastrointestinal; 1C, one-carbon metabolism.

The 120 compounds that have been approved by the FDA and which our AI-assisted workflow predicted might act as HDAC3 inhibitors are widely and diversely represented among different therapeutic areas (Figure 3). The fact that they are so broadly distributed shows the versatility of our screening method and indicates that HDAC3 inhibition could have a multitude of roles in various diseases.

The proportion of HDAC inhibitors among the identified hits was 5.0% (corresponding to six compounds), including romidepsin, vorinostat, and panobinostat, which shows that the screening process was able to recover known HDAC inhibitors. Tyrosine kinase (TK) inhibitors made up the largest group of therapeutic agents, representing 14.2% (17 compounds) of the hits. This category is especially promising since it contains already established anticancer agents, demonstrating potential opportunities for drug repurposing in the field of oncology.

Other major groups were HIV/antiviral agents and vascular and coagulation-related drugs, both accounting for 12.5 per cent (15 compounds), while RAAS/lipid-modulating agents made up 11.7 per cent (14 compounds). The fact that compounds designed for infectious diseases, cardiovascular disorders, and metabolic regulation may have previously unrecognized HDAC3 inhibitory activity is suggested by these findings.

Other defined categories were non-TK cancer therapeutics (6.7%, 8 compounds), folate/one-carbon metabolism (Folate/1C) agents (6.7%, 8 compounds), and inflammation/GI agents (6.7%, 8 compounds). There were also smaller but notable groups consisting of metabolic/PPAR-targeting drugs (4.2%, 5 compounds).

Broadly speaking, the results show that potential HDAC3 inhibitors are spread throughout a wide variety of therapeutic areas, this being a reflection of the structural and pharmacological diversity obtained through the AI-based screening approach.

### 2.3. Characterization and Docking Analysis of Predicted HDAC3 Inhibitors

While our initial screening revealed the diverse therapeutic categories from which potential HDAC3 inhibitors were identified, we next evaluated the characteristics of the highest- and lowest-ranked compounds predicted by the machine learning model. The top 10 compounds exhibited prediction scores ranging from 0.792 to 0.94 (Table 2). Notably, romidepsin, a clinically established HDAC inhibitor, achieved the highest prediction score (0.94) and showed a predicted docking score of −6.7 kcal/mol, providing an internal consistency observation for the prioritization workflow. Other highly ranked compounds included representatives from diverse therapeutic classes, such as tyrosine kinase inhibitors (imatinib and dasatinib), folate-pathway drugs (pralatrexate and methotrexate), anticoagulants (edoxaban and betrixaban), and the β3-adrenoceptor agonist mirabegron. The predicted docking score of the top-ranked compounds ranged from −6.6 to −8.3 kcal/mol.

In contrast, the bottom 10 compounds displayed substantially lower prediction scores, clustered between 0.50 and 0.52 (Table 3). Although prodrug molecules were generally excluded from the docking comparison, levorabeprazole and dexrabeprazole were retained because they undergo acid-catalyzed chemical activation rather than enzymatic metabolic activation. These compounds represented a variety of therapeutic classes, including cardiovascular drugs, antiviral agents, anticoagulants, antibiotics, proton pump inhibitors, histamine H_2_-receptor antagonists, and diagnostic radiopharmaceuticals. Their predicted docking scores ranged from −5.1 to −7.9 kcal/mol.

**Figure 4:**
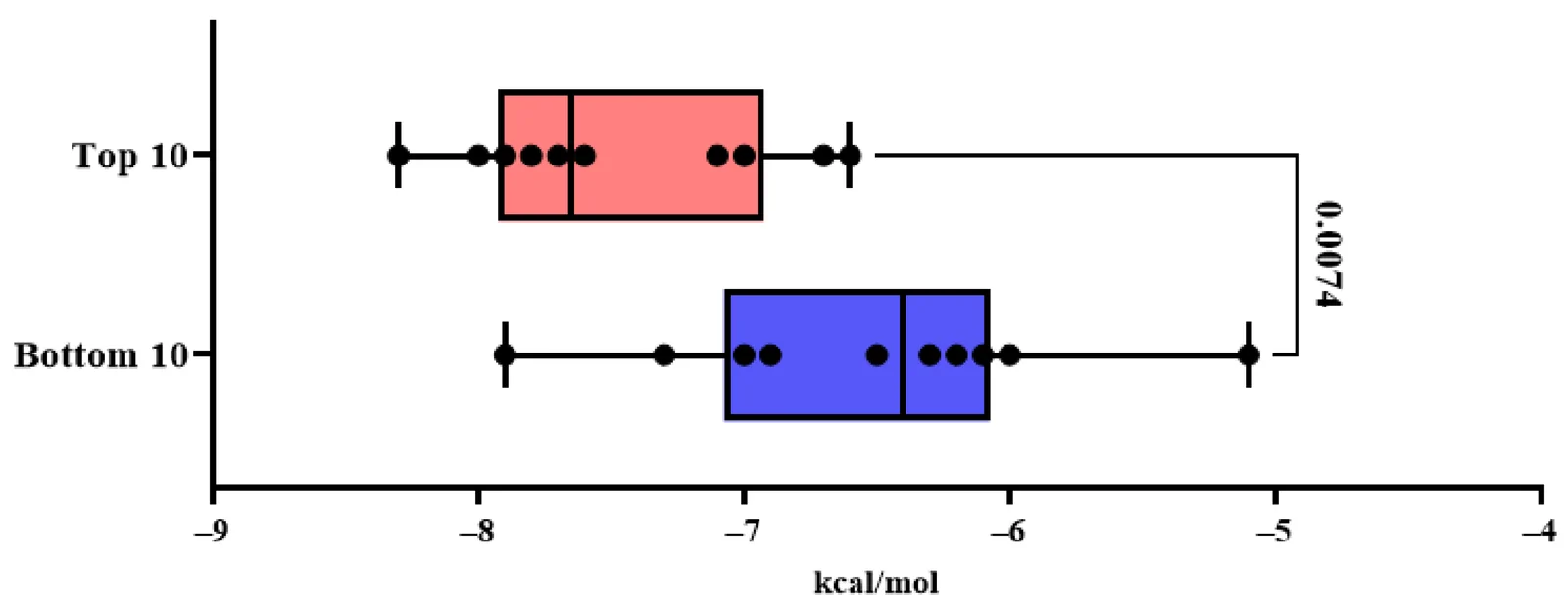
Predicted docking score comparison. Box plot showing the distribution of predicted docking scores for the top 10 and bottom 10 compounds identified by the machine learning model. More negative values indicate more favorable predicted docking scores for HDAC3 under the applied docking protocol. A significant difference in predicted docking-score distributions was observed between the two groups (Welch’s *t*-test, *p* = 0.0074).

The top 10 compounds generally exhibited more negative predicted docking score values than the bottom 10 compounds, indicating more favorable predicted docking scores. This difference in predicted docking-score distributions was statistically significant (Figure 4; *p* = 0.0074). Collectively, these results demonstrate that compounds prioritized by the machine learning model tended to exhibit more favorable predicted docking scores for HDAC3 than lower-ranked compounds.

### 2.4. Structural Overview of the HDAC3 Binding Pocket and Docking Poses of Romidepsin and Imatinib

In our prediction screening, romidepsin achieved the highest prediction score, whereas imatinib exhibited the strongest predicted docking score (−8.3 kcal/mol) among the top 10 predicted compounds. These two compounds were therefore selected for detailed docking analysis.

**Figure 5:**
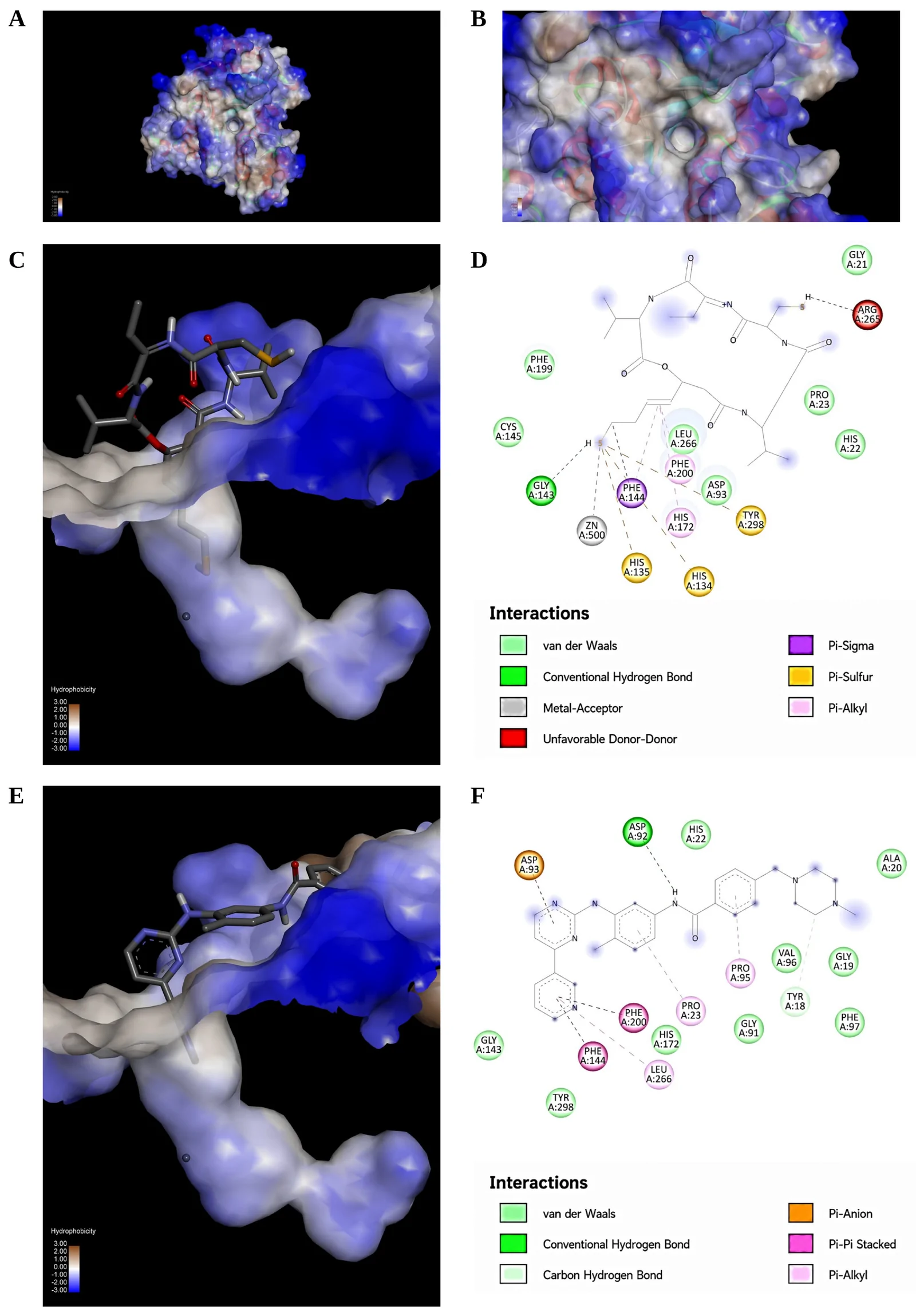
Structural visualization of the HDAC3 binding pocket and predicted docking poses of representative HDAC3 inhibitor candidates. The HDAC3 molecular surface is colored according to hydrophobicity, with blue indicating hydrophilic regions and tan/white indicating relatively hydrophobic regions. (**A**) Overall surface view of HDAC3 showing the distribution of hydrophilic and hydrophobic regions. (**B**) Enlarged view of the catalytic binding pocket. (**C**,**E**) Predicted docking poses of romidepsin and imatinib, respectively, within the HDAC3 binding tunnel. The catalytic Zn^2+^ ion is shown as a gray sphere. (**D**,**F**) Two-dimensional interaction diagrams showing the predicted interactions of romidepsin and imatinib, respectively, with residues lining the HDAC3 binding tunnel. The dashed lines indicate the specific interaction types shown in the corresponding panel legends.

Figure 5A,B display the surface characteristics of the HDAC3 binding pocket. The central tunnel is generally of intermediate hydrophobicity, there being hydrophilic areas along certain sections of the tunnel and more strongly hydrophilic regions located around the tunnel entrance. This varied surface environment offers a structural basis for accommodating ligands which have both polar and nonpolar groups.

The docking study predicted that both romidepsin and imatinib occupy the same central HDAC3 binding pocket but do so in different binding orientations (Figure 5C–F). In the case of romidepsin, it was situated close to the entrance of the tunnel, part of its macrocyclic scaffold projecting into the binding tunnel and the rest of the molecule fitting against the surrounding pocket surface. On the other hand, imatinib was found to have a more elongated orientation, with one section inserted into the tunnel and the remainder of the molecule aligned with the shape of the pocket surface. Even though the two compounds have different structures, they were both predicted to be located in the same HDAC3 binding cavity, which implies that they could interact with the catalytic channel.

The predicted interaction profile of romidepsin is shown in Figure 5C,D. Romidepsin was predicted to be positioned adjacent to the catalytic Zn^2+^ ion, with a Zn^2+^-S distance of 3.28 Å. This contact was classified as a metal-acceptor interaction in Discovery Studio Visualizer. Additional predicted interactions included a conventional hydrogen bond with GLY143, a π–sigma interaction with PHE144, π–sulfur interactions involving HIS134, HIS135, and TYR298, π–alkyl interactions with PHE200 and HIS172, and multiple van der Waals contacts with surrounding residues.

The predicted interaction profile of imatinib is shown in Figure 5E,F. Unlike romidepsin, no direct metal–acceptor interaction with the catalytic Zn^2+^ ion was predicted. Instead, imatinib was primarily stabilized by predicted π-mediated and hydrophobic interactions, including π–π stacked interactions with PHE144 and PHE200, a π–alkyl interaction with LEU266, a π–anion interaction with ASP93, a conventional hydrogen bond with ASP92, and additional van der Waals and carbon–hydrogen interactions with surrounding residues.

Overall, these predicted docking poses and interaction profiles suggest that romidepsin and imatinib occupy the same HDAC3 binding cavity while exhibiting distinct interaction patterns within the catalytic tunnel.

### 2.5. Chemical Space Analysis of Candidate HDAC3 Inhibitors

**Figure 6:**
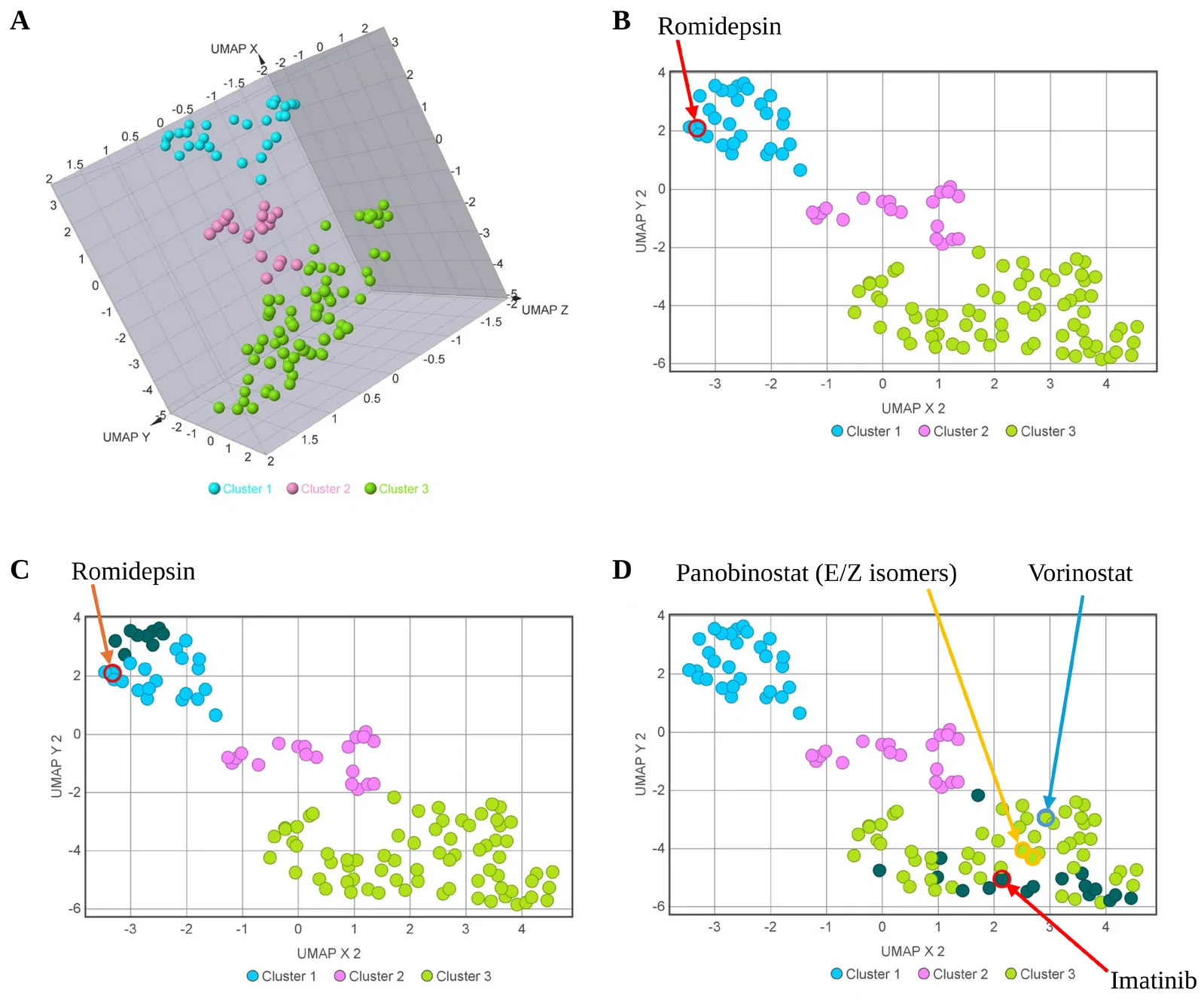
UMAP analysis of 120 candidate compounds identified from the potential HDAC3 inhibitor dataset. UMAP analysis was performed using FragFp fingerprints together with physicochemical descriptors, including molecular weight, cLogP, cLogS, hydrogen-bond acceptors, hydrogen-bond donors, polar surface area, drug-likeness, non-hydrogen atom count, rotatable bond count, and aromatic ring count. Three major chemical clusters were identified. (**A**) Three-dimensional UMAP representation. (**B**) Two-dimensional UMAP representation showing the distribution of the three major chemical clusters. Romidepsin, the highest-ranked predicted HDAC3 inhibitor, is highlighted by a red circle and is located within Cluster 1. (**C**) Distribution of nine HIV protease inhibitors (dark green), which are enriched in the romidepsin-containing region of Cluster 1. (**D**) Distribution of 17 tyrosine kinase inhibitors (TKIs; dark green). Imatinib is highlighted by a red circle, panobinostat E- and Z-isomers are highlighted by orange circles and vorinostat is highlighted by a blue circle. The panobinostat isomers are located adjacent to imatinib within the TKI-rich region of Cluster 3. The complete list of the 120 candidate HDAC3 inhibitors is provided in Supplementary Table S2A–C, and the major therapeutic characteristics of the three UMAP clusters are summarized in Table 4.

To investigate the chemical diversity of the 120 candidate compounds (Supplementary Table S2A–C), UMAP analysis was performed using FragFp fingerprints together with physicochemical descriptors. Three major chemical clusters were identified among the candidate HDAC3 inhibitors, and their major therapeutic characteristics are summarized in Table 4. The three-dimensional and two-dimensional UMAP visualizations (Figure 6A,B) demonstrate that these compounds occupy multiple distinct regions of chemical space rather than a single chemotype.

Cluster 1 contained the reference HDAC inhibitor romidepsin together with multiple HIV protease inhibitors and anticoagulants, whereas Cluster 3 contained the reference HDAC inhibitor panobinostat (E/Z) together with numerous tyrosine kinase inhibitors (TKIs), HIV reverse transcriptase inhibitors, and non-TK anticancer agents. In contrast, Cluster 2 formed an intermediate region composed primarily of folate-pathway drugs, metabolically active compounds, and RAAS/lipid-related agents.

Romidepsin was localized within Cluster 1 (Figure 6C). Notably, HIV protease inhibitors were enriched in the same region and positioned close to romidepsin, suggesting that this cluster represents a romidepsin-associated chemical space characterized by structurally complex, peptide-like compounds.

In contrast, vorinostat, panobinostat (E/Z) and imatinib were localized within the TKI-rich Cluster 3 (Figure 6D). The close spatial proximity between panobinostat and imatinib, together with other kinase inhibitors, suggests that this cluster represents a distinct HDAC inhibitor-associated chemical space that overlaps with the region occupied by kinase inhibitors.

Collectively, these findings indicate that candidate HDAC3 inhibitors occupy at least two distinct HDAC inhibitor-associated chemical spaces: a romidepsin/protease inhibitor-associated region represented by Cluster 1 and a panobinostat/TKI-associated region represented by Cluster 3. In contrast, Cluster 2 lacked a representative reference HDAC inhibitor and appeared chemically distinct from both major regions, suggesting the existence of an additional and potentially unexplored HDAC3-associated chemotype.

### 2.6. Structural Diversity and Bioavailability Assessment of the Top 15 Predicted HDAC3 Inhibitors

**Figure 7:**
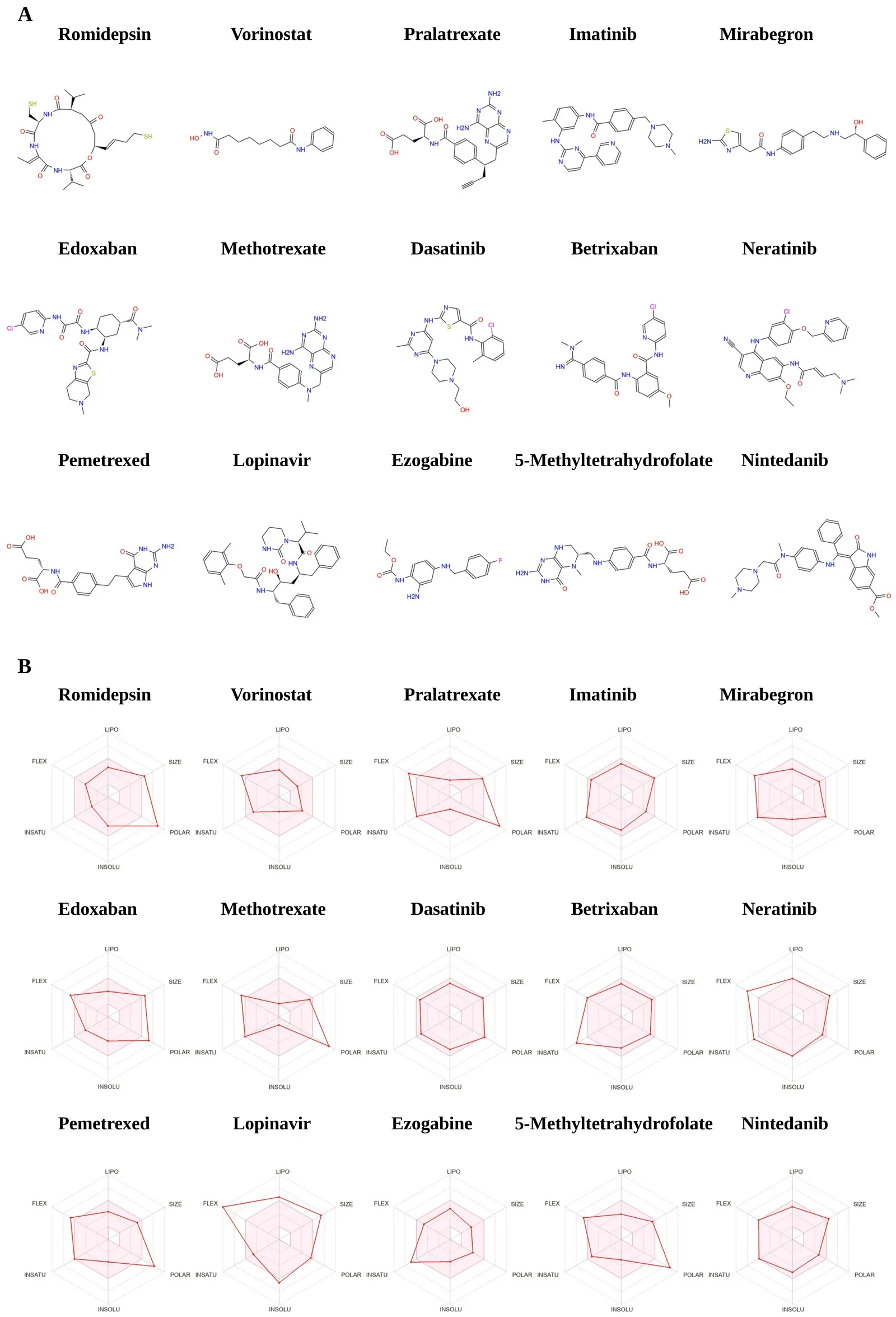
Two-dimensional chemical structures and predicted bioavailability radar profiles of the top 15 prioritized HDAC3 inhibitor candidates. (**A**) Two-dimensional chemical structures of the 15 highest-ranked compounds prioritized by the machine-learning workflow. The selected compounds encompass diverse therapeutic classes and molecular scaffolds, illustrating the structural diversity captured by the workflow. (**B**) SwissADME bioavailability radar plots of the corresponding compounds. The six axes represent lipophilicity (LIPO), molecular size (SIZE), polarity (POLAR), insolubility (INSOLU), saturation (INSATU), and flexibility (FLEX). The red polygon represents the predicted physicochemical profile of each compound, whereas the light pink region indicates the physicochemical range considered optimal for oral drug-likeness within the SwissADME model. These profiles were used for standardized comparison among the prioritized compounds and were not intended to reassess their established clinical pharmacokinetic properties.

Following the evaluation of prediction scores, docking analyses, and chemical space distribution, we next examined the two-dimensional chemical structures and predicted bioavailability profiles of the top 15 predicted HDAC3 inhibitors (Figure 7A,B). Figure 7A illustrates the structural diversity of the highest-ranked compounds, whereas Figure 7B summarizes their predicted physicochemical properties using SwissADME bioavailability radar plots.

The selected compounds include the clinically established HDAC inhibitors (romidepsin, vorinostat), the antifolates methotrexate, pralatrexate, pemetrexed, and 5-methyltetrahydrofolate, tyrosine kinase inhibitors (imatinib, dasatinib, neratinib, and nintedanib), the anticoagulants edoxaban and betrixaban, the HIV protease inhibitor lopinavir, the β3-adrenoceptor agonist mirabegron, and the potassium channel opener ezogabine. These compounds exhibit diverse molecular architectures, including macrocyclic, aromatic, heterocyclic, peptide-like, and linear scaffolds.

The marked structural diversity observed among the highest-ranked compounds indicates that the AI model did not converge on a single chemotype but instead identified multiple chemically distinct scaffolds with potential HDAC3 inhibitory activity. Together, the chemical structures and bioavailability radar plots facilitate direct comparison between molecular architecture and physicochemical properties. The tyrosine kinase inhibitors generally showed radar profiles close to the physicochemical ranges considered optimal by SwissADME. Imatinib and dasatinib remained almost entirely within the optimal ranges, whereas nintedanib showed only a slight deviation in SIZE. Neratinib showed more evident deviations in FLEX, INSATU, and SIZE. These agents, all approved for oral administration, are characterized by moderately rigid aromatic frameworks decorated with polar functionalities. Mirabegron exhibited a broadly comparable profile, with its main deviation observed in FLEX.

A second pharmacologically coherent group consisted of the anticoagulants edoxaban and betrixaban. Despite targeting different coagulation factors, their overall radar profiles were broadly similar, although the specific deviations differed. Edoxaban showed deviations in POLAR, FLEX, and SIZE, whereas betrixaban showed its main deviation in INSATU. The antifolate-related compounds, including methotrexate, pralatrexate, pemetrexed, and 5-methyltetrahydrofolate, formed another clearly recognizable group. All four compounds showed a common pattern of deviations in POLAR and FLEX, consistent with their highly functionalized structures containing multiple hydrogen-bond donors and acceptors. Despite differences in molecular size, their radar profiles were remarkably similar, reflecting their shared antifolate scaffold.

Among the remaining compounds, romidepsin showed deviations in POLAR and SIZE, consistent with its macrocyclic peptide-like structure, whereas vorinostat showed its main deviation in FLEX. Lopinavir showed clear deviations in FLEX, SIZE, and LIPO, consistent with its relatively large molecular size and complex molecular architecture. Ezogabine showed its main deviation in INSATU.

Overall, the bioavailability radar analysis revealed substantial physicochemical diversity among the top 15 predicted HDAC3 inhibitors. Together with the integrated structural and bioavailability analyses shown in Figure 7 and the chemical space distribution presented in Figure 6, these results illustrate that the highest-ranked HDAC3 candidates encompass multiple chemically distinct scaffolds while exhibiting both shared and divergent drug-like physicochemical characteristics.

### 2.7. Predicted ADMET Profiles of the Top 15 Predicted HDAC3 Inhibitors

Building upon the bioavailability assessment, we next examined the predicted absorption, distribution, metabolism, excretion, and toxicity (ADMET) profiles of the top 15 predicted HDAC3 inhibitors (Supplementary Table S3). This analysis provides a comprehensive overview of the predicted physicochemical and pharmacokinetic characteristics of the highest-ranked compounds.

Regarding absorption, predicted gastrointestinal absorption varied substantially among the compounds, ranging from 5.6% for 5-methyltetrahydrofolate to 97.8% for neratinib. The tyrosine kinase inhibitors generally exhibited high predicted gastrointestinal absorption, whereas the antifolate-related compounds, including methotrexate, pralatrexate, pemetrexed, and 5-methyltetrahydrofolate, showed considerably lower values. Predicted water solubility ranged from −2.86 to −4.51 log mol/L, indicating substantial variation in aqueous solubility across the dataset.

For distribution, all compounds exhibited negative predicted blood–brain barrier permeability (log BB) values, ranging from −0.673 to −1.892, suggesting limited blood–brain barrier penetration. Likewise, central nervous system permeability (log PS) values were consistently negative (−2.327 to −4.360). The predicted volume of distribution varied considerably, ranging from −1.259 to 1.586 log L/kg, indicating diverse predicted tissue distribution profiles.

Metabolic prediction revealed distinct patterns of cytochrome P450 inhibition among the compounds. Imatinib, betrixaban, neratinib, lopinavir, ezogabine, and nintedanib were predicted to inhibit multiple CYP isoforms. Dasatinib and mirabegron were each predicted to inhibit CYP3A4 only, whereas vorinostat was predicted to inhibit CYP1A2 alone. In contrast, romidepsin, edoxaban, and the antifolate compounds (pralatrexate, methotrexate, pemetrexed, and 5-methyltetrahydrofolate) showed no predicted CYP inhibition. No compound was predicted to inhibit CYP2D6.

Predicted total clearance ranged from −0.261 to 1.500 log ml/min/kg, demonstrating substantial variability in excretion characteristics. Among the compounds analyzed, only imatinib was predicted to be a renal OCT2 substrate.

With respect to toxicity, 14 of the 15 compounds were predicted to be Ames-negative. Ezogabine was the only compound predicted to exhibit a positive Ames toxicity prediction.

Overall, the predicted ADMET profiles revealed substantial diversity in absorption, metabolism, distribution, and excretion characteristics while consistently indicating limited predicted blood–brain barrier penetration and a low predicted mutagenic risk for most compounds. Together with the integrated structural and bioavailability analyses shown in Figure 7, these findings provide an integrated physicochemical characterization of the highest-ranked compounds identified by the AI-based screening.

### 2.8. Predicted Anticancer Cytotoxicity Profiles

Beyond the predicted ADMET characteristics (Supplementary Table S3), we next evaluated the predicted anticancer cytotoxicity profiles of the top 15 predicted HDAC3 inhibitors using CLC-Pred. CLC-Pred predicts compound activity against a broad panel of human cancer cell lines based on structural similarity to experimentally characterized active and inactive compounds. Predictions with Pa > Pi were counted as predicted active for the cell-line count analysis, consistent with CLC-Pred interpretation guidance [49]. Higher Pa values indicate a greater likelihood of experimental confirmation, and no additional Pa threshold was applied in this study.

The predicted cytotoxicity profiles varied substantially among the compounds (Table 5). Vorinostat had the largest number of cell-line predictions satisfying Pa > Pi (156), followed by methotrexate (116), imatinib (107), and pemetrexed (86). Several kinase inhibitors, including imatinib, dasatinib, neratinib, and nintedanib, also showed Pa > Pi predictions for multiple cell lines, whereas lopinavir (19), mirabegron (21), and edoxaban (24) had comparatively fewer such predictions. These counts represent the number of computational cell-line predictions satisfying Pa > Pi and do not indicate experimentally confirmed cytotoxic activity.

Average Pa values among cell-line predictions satisfying Pa > Pi ranged from 0.180 to 0.338, with romidepsin exhibiting the highest average Pa (0.338) among the compounds analyzed. Across all predictions satisfying Pa > Pi, individual Pa values ranged from 0.004 to 0.961 and Pi values ranged from 0.000 to 0.290. The cell-line predictions satisfying Pa > Pi were distributed across a wide range of tissue and organ types (Table 6). Lung-derived cell lines accounted for the largest number of predictions satisfying Pa > Pi (161), followed by blood (130) and breast (69). Multiple compounds also showed Pa > Pi predictions for cell lines derived from the colon, pancreas, stomach, kidney, skin, hematopoietic and lymphoid tissues, brain, ovary, bone, urinary tract, liver, and esophagus, illustrating the diversity of tissue origins represented among the computational predictions.

Overall, the CLC-Pred analysis demonstrated considerable diversity in the predicted anticancer cytotoxicity profiles. Together with the structural, physicochemical, and ADMET analyses presented in the preceding sections, these predictions provide a comprehensive in silico characterization of the highest-ranked compounds across multiple pharmacological properties.

## 3. Conclusions

This study established an AI-assisted workflow for prioritizing FDA-approved compounds as candidate HDAC3 inhibitors for cancer drug repurposing. The comprehensive in silico pipeline, integrating data mining, machine learning, molecular docking, chemical-space analysis, and ADMET prediction, yielded a promising set of candidates and demonstrated the potential of computational methods to accelerate FDA-approved drug repurposing.

A pivotal finding was the prioritization of 120 FDA-approved compounds as candidate HDAC3 inhibitors from a library of 1615 FDA-approved drugs. Known HDAC inhibitors already approved for cancer treatment, including romidepsin, vorinostat, and panobinostat, were recovered among the prioritized compounds, providing a useful internal consistency check for the descriptor- and fingerprint-based prioritization workflow. Independently, the Random Forest classifier achieved an accuracy of 93.1% in distinguishing the positive group from the reference comparison group using stratified 10-fold cross-validation. Together, these findings support the utility of the AI-assisted workflow for prioritizing structurally related or potentially functionally relevant compounds within the FDA-approved drug space.

Furthermore, the diverse therapeutic categories of the prioritized candidates, as illustrated in Figure 3, highlight opportunities for drug repurposing beyond conventional oncology indications. Compounds developed for other therapeutic purposes, including several tyrosine kinase inhibitors, may possess structural features compatible with HDAC3-targeted activity. The prioritization of structurally diverse candidates across multiple therapeutic classes further supports the applicability of this workflow to HDAC3-targeted drug repurposing within the FDA-approved drug space.

Our molecular docking analysis further elucidated the predicted interactions of these candidates with HDAC3. The difference in prediction scores and predicted docking-score distributions between the top- and bottom-ranked compounds (Figure 4) was consistent with the prioritization results. Structural analysis of HDAC3 revealed the presence of a narrow catalytic tunnel that guides the acetyl-lysine side chain toward the catalytic Zn^2+^ center, consistent with prior structural reports on class I HDACs (Figure 5) [50,51,52]. Our docking results predicted that both romidepsin and imatinib occupy this central tunnel, suggesting potential interference with the substrate-access pathway [53].

For romidepsin, the predicted binding mode was consistent with its known pharmacology as an FDA-approved class I HDAC inhibitor [54]. Its macrocyclic hydrophobic core was positioned within the hydrophobic interior of the tunnel, while polar functional groups were oriented toward the entrance region. Since romidepsin is a prodrug and is known to undergo intracellular reduction to generate an active thiol that coordinates Zn^2+^, our docking model shows a reasonable binding pose in which the reduced, ring-opened thiol moiety is positioned adjacent to the catalytic Zn^2+^ ion, with a predicted Zn^2+^-S distance of 3.28 Å. This predicted geometry is consistent with the established Zn^2+^-binding role of the reduced thiol and supports its placement in proximity to the catalytic metal center [53,55,56,57].

In contrast, imatinib was predicted to adopt a distinct elongated orientation stabilized primarily through aromatic and hydrophobic interactions rather than direct metal coordination. Unlike romidepsin, imatinib was not predicted to exhibit a direct metal-associated interaction with the catalytic Zn^2+^ ion. Instead, its predicted pose was characterized primarily by hydrogen bonding, π interactions, and hydrophobic contacts involving ASP92, ASP93, PHE144, PHE200, and LEU266 within the HDAC3 binding tunnel. Our initial screening identified not only imatinib but also several additional tyrosine kinase inhibitors among the top-ranked candidates, indicating that kinase inhibitors may possess an unexpected structural compatibility with the HDAC3 binding architecture.

Collectively, these findings suggest that the predicted docking poses are compatible with the accommodation of structurally diverse ligands within the HDAC3 binding pocket, including the classical macrocyclic HDAC inhibitor romidepsin and structurally distinct tyrosine kinase inhibitors. These observations support further investigation of selected tyrosine kinase inhibitors as candidates for HDAC3-targeted drug repurposing.

Interestingly, within the ML and docking-based ranking, romidepsin emerged as the top-scoring candidate (rank 1), while panobinostat, another clinically established HDAC inhibitor, was positioned at rank 80. This difference may partly reflect their distinct HDAC inhibition profiles; romidepsin preferentially targets Class I HDACs, whereas panobinostat exhibits broad inhibitory activity across multiple HDAC isoforms. However, because isoform selectivity was not directly evaluated in the present study, the difference in ranking cannot be attributed specifically to HDAC3 selectivity and may also reflect differences in molecular fingerprint representation and model scoring. Although several HDAC inhibitors, including romidepsin and panobinostat, have been approved for clinical use, no HDAC3-selective inhibitor has yet been approved. Given the high structural similarity among class I HDAC catalytic domains, achieving isoform selectivity remains a major challenge in HDAC drug discovery. Therefore, the structurally diverse compounds identified in this study may provide valuable starting scaffolds for the future development of selective HDAC3-targeted therapeutics [58,59,60].

UMAP analysis further demonstrated that the identified compounds occupy multiple distinct chemical spaces rather than converging on a single HDAC inhibitor chemotype. Notably, one chemical space was enriched with romidepsin and HIV protease inhibitors, whereas another contained panobinostat together with numerous tyrosine kinase inhibitors. These observations suggest that HDAC3 inhibition may be achieved through multiple structurally distinct molecular scaffolds and provide a broader perspective on the chemical diversity of potential HDAC3 inhibitors.

Comprehensive physicochemical and pharmacokinetic assessments, including bioavailability radar charts from SwissADME (Figure 7) and detailed ADMET profiles (Supplementary Table S3), revealed substantial variation in the predicted physicochemical and pharmacokinetic characteristics of the top 15 prioritized compounds. Although considerable variability was observed among compounds, most exhibited acceptable predicted gastrointestinal absorption, limited blood–brain barrier penetration, and low predicted mutagenicity. Metabolic profiles varied substantially, reflecting diverse patterns of CYP inhibition rather than a single common pharmacokinetic profile. These analyses provided standardized predicted profiles that facilitated comparison among structurally and pharmacologically diverse candidates. Because the evaluated compounds are FDA-approved drugs, these predictions were not intended to reassess their established clinical pharmacokinetic or safety profiles.

The CLC-Pred analysis provided a standardized computational comparison of predicted cytotoxicity profiles across the prioritized compounds. Vorinostat, methotrexate, imatinib, and pemetrexed had Pa > Pi predictions for comparatively larger numbers of cell lines, whereas romidepsin exhibited the highest average prediction probability (Pa) despite having fewer predictions satisfying Pa > Pi. The predictions satisfying Pa > Pi included cell lines from multiple tissue and organ types, including blood, lung, breast, colon, pancreas, and kidney. These results illustrate differences in the predicted cytotoxicity profiles of the prioritized compounds; however, they should be interpreted as comparative computational estimates rather than evidence of anticancer efficacy.

Integration of the structural, physicochemical, and pharmacokinetic analyses further revealed that compounds belonging to different therapeutic classes frequently converged on similar molecular design principles. Tyrosine kinase inhibitors, including imatinib, dasatinib, neratinib, and nintedanib, generally showed bioavailability radar profiles close to the physicochemical ranges considered optimal by SwissADME, with compound-specific deviations described in Section 2.6. In contrast, the antifolate compounds (methotrexate, pralatrexate, pemetrexed, and 5-methyltetrahydrofolate) formed a distinct group with highly polar physicochemical properties and comparatively limited predicted gastrointestinal absorption. Meanwhile, compounds with distinct physicochemical characteristics, such as lopinavir and vorinostat, occupied different regions of physicochemical space, illustrating that structurally diverse scaffolds can nevertheless emerge as candidate HDAC3 inhibitors. These observations suggest that molecular architecture and physicochemical balance, rather than therapeutic indication alone, are key determinants underlying the successful prioritization of potential HDAC3 inhibitors by our AI-assisted screening strategy. Although the screened compounds are FDA-approved drugs, these analyses were included to facilitate the comparative characterization of the prioritized candidates.

As discussed in the context of the HDAC3 docking analysis, the recurrent identification of tyrosine kinase inhibitors—including imatinib, dasatinib, neratinib, and nintedanib—among the highest-ranked candidates suggests that structural features originally optimized for kinase inhibition may also be compatible with the HDAC3 binding tunnel. Rather than direct coordination of the catalytic Zn^2+^ ion, these compounds appear to be stabilized primarily through hydrogen-bonding, aromatic, and hydrophobic interactions within the tunnel. This convergence suggests that pharmacophoric elements can be shared across distinct enzyme families, providing a structural basis for previously unrecognized polypharmacology. Such dual targeting may be particularly valuable in oncology, where dysregulated kinase signaling and epigenetic regulation frequently cooperate in tumor progression [61,62]. These findings therefore support further experimental evaluation of selected tyrosine kinase inhibitors as potential HDAC3-targeted drug repurposing candidates.

In summary, this study provides a proof-of-concept for an AI-assisted workflow for prioritizing FDA-approved compounds for HDAC3-targeted drug repurposing. By integrating predicted HDAC3-related prioritization with comparative computational characterization, the workflow provides a structured basis for selecting candidates for subsequent experimental investigation. Furthermore, integrating pharmacological context with structural and ADME-based classification enhances the interpretability of computational predictions and facilitates comparison of the physicochemical characteristics of structurally diverse candidates. The detailed in silico characterization of these candidates, encompassing their therapeutic categories, predicted molecular interactions, physicochemical properties, and predicted toxicity, provides a basis for experimental validation.

The limitations of this study primarily stem from its in silico nature. All computational predictions require experimental validation. Although Random Forest incorporates feature subsampling during tree construction, the number of input features was large relative to the number of compounds. Therefore, overfitting cannot be completely excluded, and independent external validation will be important in future studies. The CLC-Pred predictions are based on a training dataset, and compounds with novel structural features may exhibit unexpected biological activities. Consistent with the rapid screening objective of this workflow, molecular docking was performed using a single rigid HDAC3 receptor conformation derived from PDB 4A69. Accordingly, the predicted poses represent static binding hypotheses conditional on this crystallographic conformation and do not account for receptor flexibility or alternative binding-pocket conformations. More detailed dynamic binding behavior could be investigated in future studies using molecular dynamics simulations. Because the present workflow was specifically developed to prioritize FDA-approved compounds for drug repurposing, its applicability to other chemically distinct compound libraries has not been evaluated in the present study.

Future directions should focus on validating these promising in silico findings through rigorous in vitro and in vivo studies. This includes: (1) Experimental validation of HDAC3 inhibitory activity (e.g., enzyme assays, cellular target engagement assays); (2) Confirmation of predicted anti-cancer cytotoxicity in diverse cancer cell lines, investigating mechanisms such as apoptosis induction and cell cycle arrest; (3) Evaluation of efficacy and safety in relevant preclinical animal models; (4) Further structural optimization of the most promising leads based on detailed SAR insights derived from docking and experimental data. This multi-faceted approach will be crucial for translating these computationally identified candidates into effective novel cancer therapies.

## 4. Experimental Procedure

### 4.1. Dataset Preparation for HDAC3 Inhibitor Prediction

A dataset comprising 157 known HDAC3-related compounds with IC_50_ values of 100 nM or less was assembled from BindingDB (https://www.bindingdb.org/; accessed on 8 February 2024). These compounds, confirmed to have inhibitory activity against HDAC3, were designated as the positive group for model training. Each entry included the compound’s SMILES string, compound name or identifier, and IC_50_ value. When multiple entries existed for the same compound, only the record with the lowest IC_50_ was retained to ensure selection of the most potent variant.

To generate the reference comparison group, 157 compounds were randomly selected from a pool of 1615 FDA-approved drugs obtained from the ZINC20 database (https://zinc20.docking.org/; accessed on 19 February 2024). This reference comparison group was designed to represent the FDA-approved chemical space for drug repurposing. Random selection was performed using a random number generator, and for each compound, the SMILES structure and name were recorded.

Because the objective of this study was to develop a machine learning model based on the chemical structures of pharmacologically active compounds rather than prodrug forms, prodrugs were excluded from both the positive group and the reference comparison group before model construction. Consequently, nine prodrugs were removed from the positive group and 14 prodrugs from the reference comparison group, resulting in a final training dataset comprising 291 compounds (148 positive compounds and 143 compounds in the reference comparison group). This final balanced dataset was used for machine learning model development to identify molecular descriptor patterns associated with HDAC3 inhibition and to prioritize FDA-approved compounds for drug repurposing.

### 4.2. Molecular Descriptor and Fingerprint Calculation Using PaDEL-Descriptor

Molecular substructure fingerprints and descriptors were calculated using PaDEL-Descriptor version 2.21, a tool for computing molecular descriptors [63]. Duplicate molecules were first removed by comparing identical SMILES identifiers. The SMILES strings for the remaining unique compounds were input into PaDEL through a Python interface (Python version 3.12.4). The resulting input matrix contained 4179 PaDEL-derived molecular descriptor and fingerprint features for each compound. These features represented complementary physicochemical, topological, and molecular substructure information. The full PaDEL-derived feature matrix was used without a separate attribute-selection filter before classifier evaluation. Default PaDEL settings were retained, including salt removal, aromaticity detection, and the standardization of tautomers and nitro groups.

### 4.3. Machine Learning Model Development and Application for HDAC3 Inhibitor Prioritization

To identify potential HDAC3 inhibitors, we utilized the machine learning functionalities within the WEKA ML platform (version 3.8.6) [64]. The final training dataset comprising 291 compounds (148 known HDAC3 inhibitors and 143 compounds in the reference comparison group) was used for machine learning model development, with 4179 PaDEL-derived molecular descriptor and fingerprint features serving as input variables. Classifier performance was evaluated using stratified 10-fold cross-validation implemented in WEKA with the default random seed (seed = 1). Performance metrics, including AUC-ROC, accuracy, precision, recall, and F-measure, were calculated from the cross-validation results. We assessed the performance of five built-in WEKA classifiers: Random Forest, REPTree, Decision Table, Random Tree, and Decision Stump. The Random Forest model achieved the highest cross-validated performance among the five classifiers evaluated, with an area under the receiver operating characteristic curve (AUC-ROC) of 0.981 and an accuracy of 93.1%. After identifying the optimal model, the Random Forest classifier trained on the prodrug-excluded dataset was applied to an expanded set of 1615 FDA-approved compounds to conduct an in silico screening. This process helped prioritize FDA-approved compounds for subsequent biological validation in the context of drug repurposing. To improve model interpretability, attribute importance was evaluated using the average impurity decrease implemented in WEKA’s Random Forest classifier.

### 4.4. Molecular Docking Analysis

To explore potential binding modes of selected compounds within the HDAC3 catalytic pocket, molecular docking analysis was performed using PyRx (version 0.8; https://pyrx.sourceforge.io; accessed on 15 August 2026), a virtual screening platform that integrates AutoDock Vina (https://autodock.scripps.edu/ accessed on 15 August 2026). The crystal structure of HDAC3 (PDB ID: 4A69; https://www.rcsb.org/structure/4A69; accessed on 24 December 2024) was downloaded from the Protein Data Bank and initially prepared using UCSF ChimeraX (version 1.10.dev202412232147), a tool for interactive molecular visualization and editing [38,65]. Using the Dock Prep feature, hydrogen atoms were added (considering hydrogen-bonding networks), histidine residues were protonated appropriately, and solvent molecules were removed. The crystallographic catalytic Zn^2+^ ion was retained during receptor preparation. The prepared receptor was converted to PDBQT format in PyRx using the AutoDock “Make Macromolecule” function. In the resulting receptor PDBQT file, the catalytic Zn atom was represented as AutoDock atom type Zn. Partial-charge fields were generated automatically during PDBQT preparation. However, partial-charge values contained in the PDBQT file are not used by the standard AutoDock Vina scoring function employed in this study. Thus, no manually specified Zn charge parameter was used in the Vina scoring calculation. No specialized Zn-coordination treatment or Zn-specific docking potential was applied. The 3D structures of the selected compounds were retrieved from PubChem (https://pubchem.ncbi.nlm.nih.gov/; accessed on 24 December 2024) [66]. For molecular docking analyses, prodrug molecules were generally excluded because the objective was to evaluate the binding properties of pharmacologically active compounds. However, levorabeprazole and dexrabeprazole were retained because they undergo acid-catalyzed chemical activation rather than enzymatic metabolic activation. Accordingly, the top 10 non-prodrug compounds and the bottom 10 compounds (including levorabeprazole and dexrabeprazole) ranked according to the machine learning prediction scores were selected for docking analysis. For subsequent structural visualization and protein–ligand interaction analyses, the top 15 non-prodrug compounds were selected. These compounds were imported into PyRx along with the prepared HDAC3 receptor structure. Docking simulations were performed using AutoDock Vina with default search parameters, except for the manually defined docking search space. The docking search space was centered at X = 37.2, Y = 57.1, and Z = 22.7, with grid box dimensions of 29.8 × 29.2 × 31.0 Å. These settings were selected to encompass the entire HDAC3 catalytic tunnel, including the catalytic Zn^2+^ site and the surrounding substrate-binding pocket [67,68]. The most negative AutoDock Vina docking score (kcal/mol) was recorded as the primary comparative score for each compound.

Following the docking simulations, the protein–ligand complexes were further examined for interactions within the HDAC3 active site using Discovery Studio Visualizer (version 2024), a molecular graphics tool designed for analyzing molecular assemblies [69]. Protein–ligand interactions, including hydrogen bonds, hydrophobic interactions, π interactions, and metal-associated interactions, were analyzed using Discovery Studio Visualizer.

### 4.5. UMAP-Based Clustering Analysis

A total of 120 candidate HDAC3 inhibitors identified from the WEKA screening were subjected to clustering analysis using DataWarrior (version 06.05.04; https://openmolecules.org/datawarrior/; accessed on 14 June 2026), an interactive chemistry-aware data analysis and visualization software. Unlike the docking and structure-based analyses, all 120 candidate compounds, including prodrug molecules, were included in the UMAP analysis to visualize the overall chemical space represented by the machine learning predictions. The chemical structures were imported in SMILES format. Fingerprint-based similarity analysis was performed using the FragFp descriptor implemented in DataWarrior. UMAP dimensionality reduction was subsequently applied using FragFp fingerprints together with physicochemical descriptors to visualize the distribution of compounds in chemical space.

Compounds were grouped according to their distribution in the UMAP projection, and three major chemical clusters were identified for subsequent analyses. Romidepsin, the highest-scoring predicted HDAC3 inhibitor, was located within Cluster 1 and was selected as the primary reference compound because it represents a clinically established class I HDAC inhibitor. Panobinostat, another clinically established HDAC inhibitor, was identified within Cluster 3 and was included as a representative reference compound for comparison with romidepsin. The positions of these reference compounds within the UMAP projection were used to evaluate the structural relationships between clinically established HDAC inhibitors and the remaining predicted compounds.

### 4.6. ADMET Analysis

ADMET (absorption, distribution, metabolism, excretion, and toxicity) properties are essential to evaluate during drug discovery to assess pharmacological characteristics before in vitro testing. The SMILES strings of the top 15 non-prodrug compounds were analyzed using multiple in silico prediction tools to generate standardized physicochemical, pharmacokinetic, and predicted cytotoxicity profiles for comparative characterization. The SMILES used for these analyses were obtained from the ZINC20 database, and the corresponding ZINC identifiers and exact SMILES used for all 15 compounds are provided in Supplementary Table S4 to ensure reproducibility. The SwissADME web tool (https://www.swissadme.ch/; accessed on 14 June 2026) was used to predict detailed physicochemical and pharmacokinetic parameters, which were visualized as bioavailability radar charts to evaluate drug likeness [70]. pkCSM (https://biosig.lab.uq.edu.au/pkcsm/; accessed on 14 June 2026) provided predictions of overall pharmacokinetic behavior, contributing to the comprehensive ADMET profiles [71]. Cell Line Cytotoxicity Predictor (CLC-Pred 2.0; https://www.way2drug.com/clc-pred/; accessed on 15 June 2026), a specialized web-based tool for the in silico prediction of the cytotoxic effects of chemical and natural compounds on various human cell lines, was employed to estimate potential cytotoxic effects across various cancer cell lines, facilitating comparison of predicted cytotoxicity profiles across the prioritized compounds [49]. Relevant ADMET parameters were collected from these analyses, encompassing aspects of absorption (e.g., water solubility, gastrointestinal absorption), distribution (e.g., blood–brain barrier permeability, volume of distribution), metabolism (e.g., cytochrome P450 enzyme inhibition profiles), excretion (e.g., total clearance), and toxicity (e.g., Ames toxicity). These parameters were used to facilitate comparative characterization of the prioritized compounds.

## Figures and Tables

**Table 1:** Performance Metrics of Machine Learning Classifiers.

Rank	Model Name	AUC-ROC	TP Rate	FP Rate	Accuracy	Precision	F-Measure	Time (s)
1	Random Forest	0.981	0.959	0.098	0.931	0.910	0.934	0.490
2	Decision Table	0.956	0.966	0.126	0.921	0.888	0.926	4.810
3	REP Tree	0.919	0.932	0.133	0.900	0.879	0.905	0.260
4	Random Tree	0.866	0.885	0.154	0.866	0.856	0.870	0.010
5	Decision Stump	0.813	0.912	0.308	0.804	0.754	0.826	0.070

**Table 2:** Compounds with the top prediction scores.

Compound Name	Prediction Score	Drug Category	Predicted Docking Score
Romidepsin (active reduced form)	0.94	HDAC inhibitor; active metabolite/form	−6.7
Vorinostat	0.85	HDAC inhibitor	−7.0
Pralatrexate (4R,25S isomer)	0.84	Folates and folate-pathway drugs	−7.6
Pralatrexate (4S,25S isomer)	0.84	Folates and folate-pathway drugs	−8.0
Imatinib	0.83	Tyrosine kinase inhibitor	−8.3
Mirabegron	0.827	β3-adrenoceptor agonist; overactive bladder therapy	−7.7
Edoxaban	0.82	Anticoagulant	−7.1
Methotrexate	0.81	Antifolate antimetabolite	−7.8
Dasatinib	0.80	Tyrosine kinase inhibitor	−7.9
Betrixaban	0.792	Anticoagulant	−6.6

**Table 3:** Compounds with the Lowest Prediction Scores.

Compound Name	Prediction Score	Drug Category	Predicted Docking Score
Rivaroxaban	0.52	Anticoagulant	−7.0
Darunavir	0.52	Antiviral agent	−7.3
Candesartan	0.52	Cardiovascular drug	−7.9
(R)-Dobutamine	0.52	Cardiovascular drug	−6.9
Florbetapir	0.508	Diagnostic radiopharmaceutical/β-amyloid PET imaging agent	−6.2
Florbetapir (AV-45 stereoisomer)	0.508	Diagnostic radiopharmaceutical/β-amyloid PET imaging agent	−6.1
Levorabeprazole [(S)-rabeprazole]	0.502	Proton pump inhibitor; prodrug	−6.5
Dexrabeprazole [(R)-rabeprazole]	0.502	Proton pump inhibitor; prodrug	−6.3
Ranitidine	0.50	Histamine H2-receptor antagonist; acid-suppressive agent	−5.1
Cefepime	0.50	Antibiotic and antimicrobial	−6.0

**Table 4:** Summary of the therapeutic characteristics of the three UMAP clusters.

Cluster	Reference HDAC Inhibitor	Enriched Therapeutic Categories	Representative Compounds
Cluster 1	Romidepsin	HIV protease inhibitors; Vascular/coagulation	Romidepsin, Ritonavir, Lopinavir, Edoxaban, Argatroban
Cluster 2	None	Folate/1C; Metabolic/PPAR; RAAS/lipid	Methotrexate, Pralatrexate, Pemetrexed, Rosiglitazone, Pioglitazone
Cluster 3	Vorinostat, Panobinostat (E/Z)	TK inhibitors; HIV reverse transcriptase inhibitors; Non-TK cancer	Vorinostat, Panobinostat (E/Z), Imatinib, Afatinib, Ponatinib, Delavirdine

**Table 5:** Compound-Specific Summary of Predicted Active Cell Lines and Pa/Pi Values.

Compound Name	Predicted Active Cell Lines	Average Pa (Active Predictions)	Pa Range	Pi Range
Vorinostat	156	0.301814	0.009–0.858	0.001–0.233
Methotrexate	116	0.308888	0.007–0.875	0.001–0.255
Imatinib	107	0.286486	0.057–0.961	0.000–0.248
Pemetrexed	86	0.273628	0.004–0.652	0.003–0.259
Betrixaban	68	0.250868	0.040–0.454	0.023–0.238
Pralatrexate stereoisomer R	66	0.295197	0.020–0.680	0.002–0.221
Dasatinib	59	0.323864	0.110–0.819	0.001–0.254
Neratinib	58	0.277172	0.033–0.859	0.003–0.290
Ezogabine	53	0.229245	0.050–0.545	0.005–0.254
5-Methyltetrahydrofolate	47	0.267106	0.005–0.793	0.002–0.250
Romidepsin active form	38	0.338026	0.069–0.813	0.003–0.227
Nintedanib	31	0.279097	0.085–0.646	0.012–0.232
Edoxaban	24	0.248958	0.060–0.497	0.011–0.285
Mirabegron	21	0.226429	0.083–0.327	0.011–0.267
Lopinavir	19	0.180316	0.029–0.354	0.004–0.221

Predicted active cell lines were defined as those with Pa > Pi. Average Pa and Pa/Pi ranges were calculated only for predictions satisfying this criterion.

**Table 6:** Distribution of Predicted Active Cell Lines by Tissue/Organ Type. Only Tissue/Organ types with 10 or more predicted active compounds are displayed.

Tissue/Organ	Number of Compounds	Number of Active Cell Lines
Blood	15	130
Breast	15	69
Lung	14	161
Colon	14	56
Pancreas	14	45
Stomach	14	43
Kidney	14	41
Skin	13	60
Haematopoietic and lymphoid tissue	13	54
Brain	13	32
Ovarium	12	39
Bone	11	25
Urinary tract	11	24
Liver	11	19
Esophagus	10	15

## Data Availability

All data generated or analyzed during this study are either included in this published article (and its Supplementary Information Files) or available from the authors upon reasonable request. The original compound libraries were accessed via BindingDB, ZINC20, and PubChem.

## Supplementary Materials

The following supporting information can be downloaded at: https://www.mdpi.com/article/10.3390/biology15181570/s1, Supplementary Table S1: Top 30 molecular features ranked by Random Forest feature importance (average impurity decrease) implemented in WEKA; Supplementary Table S2A: Compounds assigned to UMAP Cluster 1; Supplementary Table S2B: Compounds assigned to UMAP Cluster 2; Supplementary Table S2C: Compounds assigned to UMAP Cluster 3; Supplementary Table S3: Predicted ADMET profiles of the top 15 predicted HDAC3 inhibitors; Supplementary Table S4: ZINC identifiers and exact SMILES used for the top 15 predicted HDAC3 inhibitor candidates.

## Abbreviations

ADMET, absorption, distribution, metabolism, excretion, and toxicity; AI, artificial intelligence; ALL, acute lymphoblastic leukemia; AML, acute myeloid leukemia; AUC-ROC, area under the receiver operating characteristic curve; BRD3308, 4-acetamido-N-(2-amino-4-fluorophenyl)benzamide; CLC-Pred, Cell Line Cytotoxicity Predictor; CLL, chronic lymphocytic leukemia; CNS, central nervous system; CYP, cytochrome P450; DLBCL, diffuse large B-cell lymphoma; FDA, Food & Drug Administration; FragFp, Fragment Fingerprint; GI, gastrointestinal; HAT, histone acetyltransferase; HDAC, histone deacetylase; HDACi, histone deacetylase inhibitor; IAP*, Invariant Accuracy of Prediction; IC_50_, half-maximal inhibitory concentration; Log BB, blood–brain barrier; LogP, octanol-water partition coefficient; ML, machine learning; NAD^+^, nicotinamide adenine dinucleotide; NCoR, nuclear receptor corepressor; Pa, predicted active; PCA, principal component analysis; PDB, Protein Data Bank; Pi, predicted inactive; PyRx, Python Prescription; REPTree, reduced error pruning tree; RGFP966, (2E)-N-(2-amino-4-fluorophenyl)-3-[1-(3-phenyl-2-propen-1-yl)-1H-pyrazol-4-yl]-2-propenamide; ROC, receiver operating characteristic; SAR, structure–activity relationship; SMILES, Simplified Molecular-Input Line-Entry System; SMRT, silencing mediator of retinoic acid and thyroid hormone receptor; SwissADME, Swiss absorption, distribution, metabolism, excretion; TCGA, The Cancer Genome Atlas; TKI, tyrosine kinase inhibitor; t-SNE, t-distributed stochastic neighbor embedding; WEKA, Waikato Environment for Knowledge Analysis; Wnt, “Wingless” and “Int-1,” a family of signaling proteins critical for cell communication, development, and tissue maintenance.
