# Supplementary material for: An AI-Assisted Workflow for Rapid Prioritization of FDA-Approved Drugs as HDAC3 Inhibitor Candidates for Drug Repurposing
Source: Biology (Basel). 2026 Sep 8;15(18):1570. doi: 10.3390/biology15181570 (PMC13603992; doi:10.3390/biology15181570)
Supplement: Supplementary file 1 [file biology-15-01570-s001.zip › biology-4483703-supplementary.pdf]

biology MDPI

Supplementary Materials

Supplementary Table S1. Top 30 molecular features ranked by Random Forest feature importance (average impurity decrease) implemented in WEKA.

Rank	Descriptor	Relative importance	Nodes using descriptor
1	PJI2	1.00	1
2	MPC04	1.00	1
3	VR1_B(p)	1.00	1
4	MWC09	1.00	1
5	JGT	1.00	1
6	SpPosLog_Dt	1.00	1
7	ATS4p	1.00	1
8	Chi_Dt	1.00	1
9	s4_phSize	1.00	1
10	GGI2	1.00	1
11	J_A	1.00	1
12	SM3_B(e)	1.00	1
13	nR06	1.00	1
14	X4Av	1.00	1
15	X3	1.00	1
16	RFD	1.00	1
17	CID	1.00	1
18	SpMax5_Bh(p)	1.00	1
19	ATS7p	1.00	1
20	P_VSA_m_1	0.99	1
21	GATS3i	0.99	1
22	s34_phRelSize	0.99	1
23	SM2_Dz(Z)	0.99	1
24	SpMax_EA	0.99	1
25	J_X	0.97	1
26	Eig04_AEA(dm)	0.97	1
27	GATS2p	0.97	1
28	TRS	0.96	2
29	P_VSA_s_2	0.96	2
30	SM15_EA(dm)	0.92	1

Descriptor names correspond to the original PaDEL-Descriptor nomenclature. Relative importance values were calculated using the Random Forest attribute importance function in WEKA based on the average impurity decrease.

Supplementary Table S2A. Compounds assigned to UMAP Cluster 1.

ZINC ID	Compound	Figure 3 Category	Therapeutic class
ZINC000100371951	Romidepsin active form / reduced romidepsin	HDAC inhibitors	HDAC inhibitor
ZINC000306122005	Romidepsin parent form / disulfide romidepsin (E-isomer)	HDAC inhibitors	HDAC inhibitor
ZINC000254134439	Romidepsin parent form / disulfide romidepsin (Z-isomer)	HDAC inhibitors	HDAC inhibitor
ZINC000085537014	Cobicistat	HIV/antiviral	PK booster / CYP3A inhibitor
ZINC000003955219	Darunavir	HIV/antiviral	HIV protease inhibitor
ZINC000022448696	Indinavir	HIV/antiviral	HIV protease inhibitor
ZINC000003951740	Lopinavir	HIV/antiviral	HIV protease inhibitor
ZINC000003833846	Nelfinavir	HIV/antiviral	HIV protease inhibitor
ZINC000013831130	Raltegravir	HIV/antiviral	HIV integrase inhibitor
ZINC000003944422	Ritonavir	HIV/antiviral	HIV protease inhibitor
ZINC000029416466	Saquinavir stereoisomer (CIP: 8S; 10R; 15R; 19R; 21S; 32S)	HIV/antiviral	HIV protease inhibitor
ZINC000026985532	Saquinavir stereoisomer (CIP: 8S; 10R; 15S; 19R; 21S; 32S)	HIV/antiviral	HIV protease inhibitor
ZINC000026664090	Saquinavir stereoisomer (CIP: 8S; 10S; 15R; 19R; 21S; 32S)	HIV/antiviral	HIV protease inhibitor
ZINC000003914596	Saquinavir stereoisomer (CIP: 8S; 10S; 15S; 19R; 21S; 32S)	HIV/antiviral	HIV protease inhibitor
ZINC000003812863	Lisinopril	RAAS/lipid	ACE inhibitor
ZINC000003812306	Moexipril	RAAS/lipid	ACE inhibitor
ZINC000003801163	Quinapril	RAAS/lipid	ACE inhibitor
ZINC000003875259	Valsartan	RAAS/lipid	Angiotensin II receptor blocker (ARB)
ZINC000150338698	Capreomycin	Other	Antitubercular antibiotic
ZINC000003830405	Cefazolin	Other	Cephalosporin antibiotic
ZINC000004215234	Cefditoren pivoxil	Other	Cephalosporin antibiotic
ZINC000003871923	Cefepime	Other	Cephalosporin antibiotic
ZINC000002005305	5-Methyltetrahydrofolate	Folate/1C	Folate derivative
ZINC000009212428	Leucovorin / Folinic acid stereoisomer (CIP: 12R; 22S)	Folate/1C	Folate derivative
ZINC000009212427	Levoleucovorin / (6S)-Folinic acid	Folate/1C	Folate derivative
ZINC000003807172	Argatroban	Vascular/coagulation	Direct thrombin inhibitor
ZINC000043200832	Edoxaban	Vascular/coagulation	Factor Xa inhibitor
ZINC000169746649	Bortezomib	Non-TK cancer	Proteasome inhibitor
ZINC000014210876	Eluxadoline	Inflammation/GI	Mixed opioid receptor modulator

This table lists compounds assigned to the indicated UMAP cluster. Compounds are presented with their ZINC identifiers, Figure 3 therapeutic categories, and detailed therapeutic classes. HDAC inhibitors are listed first; all other therapeutic categories are ordered by the number of compounds within the cluster.

Supplementary Table S2B. Compounds assigned to UMAP Cluster 2.

ZINC ID	Compound	Figure 3 Category	Therapeutic class
ZINC000003920719	Atorvastatin stereoisomer (CIP: 33R; 36R)	RAAS/lipid	HMG-CoA reductase inhibitor / statin
ZINC000011615927	Atorvastatin stereoisomer (CIP: 33R; 36S)	RAAS/lipid	HMG-CoA reductase inhibitor / statin
ZINC000011615926	Atorvastatin stereoisomer (CIP: 33S; 36R)	RAAS/lipid	HMG-CoA reductase inhibitor / statin
ZINC000011615928	Atorvastatin stereoisomer (CIP: 33S; 36S)	RAAS/lipid	HMG-CoA reductase inhibitor / statin
ZINC000029319828	Eprosartan	RAAS/lipid	Angiotensin II receptor blocker (ARB)
ZINC000003792417	Sacubitril	RAAS/lipid	Neprilysin inhibitor
ZINC000008577218	Folic Acid	Folate/1C	Folate
ZINC000001529323	Methotrexate	Folate/1C	Antifolate
ZINC000001540998	Pemetrexed	Folate/1C	Antifolate
ZINC000011616925	Pralatrexate stereoisomer (CIP: 4R; 25S)	Folate/1C	Antifolate
ZINC000001536109	Pralatrexate stereoisomer (CIP: 4S; 25S)	Folate/1C	Antifolate
ZINC000100052681	Iloprost stereoisomer (CIP: 5R; 7R; 11R; 12S; 15S; 35R; 9=10:E; 17=18:Z)	Vascular/coagulation	Prostacyclin analog
ZINC000100052688	Iloprost stereoisomer (CIP: 5R; 7S; 11R; 12S; 15S; 35R; 9=10:E; 17=18:Z)	Vascular/coagulation	Prostacyclin analog
ZINC000100052685	Iloprost stereoisomer (CIP: 5S; 7R; 11R; 12S; 15S; 35R; 9=10:E; 17=18:Z)	Vascular/coagulation	Prostacyclin analog
ZINC000100052691	Iloprost stereoisomer (CIP: 5S; 7S; 11R; 12S; 15S; 35R; 9=10:E; 17=18:Z)	Vascular/coagulation	Prostacyclin analog
ZINC000003806104	Tirofiban	Vascular/coagulation	GP IIb/IIIa inhibitor
ZINC000000968327	(R)-Pioglitazone	Metabolic/PPAR	PPARγ agonist
ZINC000000968330	(R)-Rosiglitazone	Metabolic/PPAR	PPARγ agonist
ZINC000000968326	(S)-Pioglitazone	Metabolic/PPAR	PPARγ agonist
ZINC000000968328	(S)-Rosiglitazone	Metabolic/PPAR	PPARγ agonist

This table lists compounds assigned to the indicated UMAP cluster. Compounds are presented with their ZINC identifiers, Figure 3 therapeutic categories, and detailed therapeutic classes. HDAC inhibitors are listed first; all other therapeutic categories are ordered by the number of compounds within the cluster.

Supplementary Table S2C. Compounds assigned to UMAP Cluster 3.

ZINC ID	Compound	Figure 3 Category	Therapeutic class
ZINC000022010649	Panobinostat (E-isomer)	HDAC inhibitors	HDAC inhibitor
ZINC000051951647	Panobinostat (Z-isomer)	HDAC inhibitors	HDAC inhibitor
ZINC000001543873	Vorinostat	HDAC inhibitors	HDAC inhibitor
ZINC000000003911	(R)-Dobutamine	Other	β-adrenergic receptor agonist
ZINC000094566093	(R)-Doxazosin	Other	α1-adrenergic receptor antagonist
ZINC000000057278	(S)-Dobutamine	Other	β-adrenergic receptor agonist
ZINC000094566092	(S)-Doxazosin	Other	α1-adrenergic receptor antagonist
ZINC000001542392	Alfuzosin	Other	α1-adrenergic receptor antagonist
ZINC000014768621	Chlorhexidine	Other	Antiseptic
ZINC000012503187	Conivaptan	Other	Vasopressin receptor antagonist
ZINC000003830635	Deferoxamine	Other	Iron chelator
ZINC000000016154	Ezogabine / Retigabine	Other	Kv7 potassium channel opener
ZINC000043207237	Florbetapir	Other	Amyloid PET tracer
ZINC000145808877	Florbetapir stereoisomer / AV-45 stereoisomer	Other	Amyloid PET tracer
ZINC000001996784	Mirabegron	Other	β3-adrenergic receptor agonist
ZINC000000538065	Nefazodone	Other	Serotonin antagonist and reuptake inhibitor
ZINC000001530775	Pentamidine	Other	Antiprotozoal
ZINC000053084692	Safinamide	Other	MAO-B inhibitor
ZINC000003806063	Silodosin	Other	α1A-adrenergic receptor antagonist
ZINC000043100953	Tedizolid phosphate	Other	Oxazolidinone antibiotic prodrug
ZINC000084758235	Telotristat	Other	Tryptophan hydroxylase inhibitor
ZINC000001542113	Vilazodone	Other	SSRI / 5-HT1A partial agonist
ZINC000003976838	Afatinib	TK inhibitors	Tyrosine kinase inhibitor (TKI)
ZINC000003816287	Axitinib (E-isomer)	TK inhibitors	Tyrosine kinase inhibitor (TKI)
ZINC000011616882	Axitinib (Z-isomer)	TK inhibitors	Tyrosine kinase inhibitor (TKI)
ZINC000148723177	Brigatinib	TK inhibitors	Tyrosine kinase inhibitor (TKI)
ZINC000070466416	Cabozantinib	TK inhibitors	Tyrosine kinase inhibitor (TKI)
ZINC000096272772	Ceritinib	TK inhibitors	Tyrosine kinase inhibitor (TKI)
ZINC000003986735	Dasatinib	TK inhibitors	Tyrosine kinase inhibitor (TKI)
ZINC000035328014	Ibrutinib	TK inhibitors	BTK inhibitor / tyrosine kinase inhibitor
ZINC000019632618	Imatinib	TK inhibitors	Tyrosine kinase inhibitor (TKI)
ZINC000003816292	Lenvatinib	TK inhibitors	Tyrosine kinase inhibitor (TKI)
ZINC000003916214	Neratinib	TK inhibitors	Tyrosine kinase inhibitor (TKI)
ZINC000006716957	Nilotinib	TK inhibitors	Tyrosine kinase inhibitor (TKI)
ZINC000100014909	Nintedanib	TK inhibitors	Tyrosine kinase inhibitor (TKI)
ZINC000098023177	Osimertinib	TK inhibitors	Tyrosine kinase inhibitor (TKI)
ZINC000036701290	Ponatinib	TK inhibitors	Tyrosine kinase inhibitor (TKI)
ZINC000006745272	Regorafenib	TK inhibitors	Tyrosine kinase inhibitor (TKI)
ZINC000001493878	Sorafenib	TK inhibitors	Tyrosine kinase inhibitor (TKI)

ZINC000030691754	Betrixaban	Vascular/coagulation	Factor Xa inhibitor
ZINC000001538857	Bosentan	Vascular/coagulation	Endothelin receptor antagonist
ZINC000003943279	Dabigatran Etexilate	Vascular/coagulation	Direct thrombin inhibitor
ZINC000000596731	Dofetilide	Vascular/coagulation	Class III antiarrhythmic
ZINC000011679756	Eltrombopag	Vascular/coagulation	Thrombopoietin receptor agonist
ZINC000043202140	Macitentan	Vascular/coagulation	Endothelin receptor antagonist
ZINC000003819392	Riociguat	Vascular/coagulation	Soluble guanylate cyclase stimulator
ZINC000003964126	Rivaroxaban	Vascular/coagulation	Factor Xa inhibitor
ZINC000003990451	Selexipag	Vascular/coagulation	Prostacyclin receptor agonist
ZINC000028957444	Ticagrelor	Vascular/coagulation	P2Y12 receptor antagonist
ZINC000072318121	Abemaciclib	Non-TK cancer	CDK4/6 inhibitor
ZINC000068247389	Copanlisib	Non-TK cancer	PI3K inhibitor
ZINC000040430143	Olaparib	Non-TK cancer	PARP inhibitor
ZINC000003938686	Palbociclib	Non-TK cancer	CDK4/6 inhibitor
ZINC000072316335	Ribociclib	Non-TK cancer	CDK4/6 inhibitor
ZINC000068202099	Sonidegib	Non-TK cancer	SMO inhibitor
ZINC000052509366	Vemurafenib	Non-TK cancer	RAF inhibitor
ZINC000004618208	(R)-Rabeprazole / Dexrabeprazole	Inflammation/GI	Proton pump inhibitor
ZINC000001530935	(S)-Rabeprazole / Levorabeprazole	Inflammation/GI	Proton pump inhibitor
ZINC000001843099	Alibendol	Inflammation/GI	GI smooth muscle relaxant / choleretic
ZINC000003952881	Balsalazide	Inflammation/GI	5-ASA prodrug
ZINC000001530728	Ranitidine	Inflammation/GI	H2 receptor antagonist
ZINC000003831490	Sulfasalazine	Inflammation/GI	5-ASA prodrug / DMARD
ZINC000018516586	Delavirdine	HIV/antiviral	HIV NNRTI
ZINC000000602632	Etravirine	HIV/antiviral	HIV NNRTI
ZINC000001554274	Rilpivirine	HIV/antiviral	HIV NNRTI
ZINC000100055899	Tenofovir Alafenamide	HIV/antiviral	HIV nucleotide reverse transcriptase inhibitor prodrug
ZINC000014210642	Azilsartan	RAAS/lipid	Angiotensin II receptor blocker (ARB)
ZINC000003782818	Candesartan	RAAS/lipid	Angiotensin II receptor blocker (ARB)
ZINC000004097427	Candesartan cilexetil stereoisomer (CIP: 14R)	RAAS/lipid	ARB prodrug
ZINC000004074875	Candesartan cilexetil stereoisomer (CIP: 14S)	RAAS/lipid	ARB prodrug
ZINC000000537795	Glipizide	Metabolic/PPAR	Sulfonylurea

This table lists compounds assigned to the indicated UMAP cluster. Compounds are presented with their ZINC identifiers, Figure 3 therapeutic categories, and detailed therapeutic classes. HDAC inhibitors are listed first; all other therapeutic categories are ordered by the number of compounds within the cluster.

Supplementary Table S3. Predicted ADMET Profiles of Top 15 Predicted HDAC3 Inhibitors.

Properties	Parameters	Romidepsin active form	Vorinostat	Pralatrexate stereoisomer (CIP: 4R; 25S)	Imatinib	Mirabegron	Edoxaban	Methotrexate	Dasatinib	Betrixaban	Neratinib	Pemetrexed	Lopinavir	Ezogabine / Retigabine	5-Methyltetrahydrofolate	Nintedanib
Absorption	Water Solubility (log mol/L)	-3.539	-3.153	-2.929	-3.379	-3.908	-3.419	-2.911	-3.619	-4.152	-4.173	-2.907	-4.509	-3.439	-2.857	-4.049
	Gastrointestinal (%)	48.562	88.314	16.711	93.146	74.322	71.248	15.475	73.447	78.615	97.830	15.709	64.416	90.326	5.645	83.742
Distribution	Blood Brain Barrier (log BB)	-1.033	-0.679	-1.647	-1.418	-0.920	-1.148	-1.775	-1.576	-1.417	-1.568	-1.592	-0.673	-0.908	-1.892	-0.738
	Central Nervous System (log PS)	-2.923	-2.825	-4.360	-2.395	-2.791	-3.391	-3.821	-3.161	-2.470	-2.488	-4.034	-2.856	-2.327	-4.212	-2.510
	Volume of Distribution (log L/kg)	-1.259	0.120	-0.419	1.586	0.850	-0.444	-0.467	0.708	-0.079	0.989	-0.832	-0.047	-0.034	-0.565	0.721
Metabolism	CYP1A2 inhibitor	No	Yes	No	No	No	No	No	No	No	No	No	No	Yes	No	No
	CYP2C19 inhibitor	No	No	No	No	No	No	No	No	Yes	Yes	No	Yes	Yes	No	Yes
	CYP2C9 inhibitor	No	No	No	Yes	No	No	No	No	Yes	No	No	Yes	Yes	No	Yes
	CYP2D6 inhibitor	No	No	No	No	No	No	No	No	No	No	No	No	No	No	No
	CYP3A4 inhibitor	No	No	No	Yes	Yes	No	No	Yes	Yes	Yes	No	Yes	No	No	Yes
Excretion	Total Clearance (log ml/min/kg)	-0.261	0.434	0.292	0.758	1.500	0.451	0.416	0.472	0.260	0.749	0.328	0.486	-0.056	0.161	0.876
	Renal OCT2 substrate	No	No	No	Yes	No	No	No	No	No	No	No	No	No	No	No
Toxicity	AMES Toxicity	No	No	No	No	No	No	No	No	No	No	No	No	Yes	No	No

Predicted absorption, distribution, metabolism, excretion, and toxicity (ADMET) properties of the top 15 predicted HDAC3 inhibitors are summarized. Parameters include water solubility (log mol/L), gastrointestinal (GI) absorption (%), blood–brain barrier permeability (log BB), central nervous system permeability (log PS), volume of distribution (log L/kg), predicted inhibition of major cytochrome P450 (CYP) isoforms (CYP1A2, CYP2C19, CYP2C9, CYP2D6, and CYP3A4), total clearance (log ml/min/kg), renal OCT2 substrate status, and Ames toxicity prediction. ADMET properties were predicted using SwissADME and pkCSM.

Supplementary Table S4. ZINC identifiers and exact SMILES used for the top 15 predicted HDAC3 inhibitor candidates.

ZINC ID	Compound Annotation	SMILES
**ZINC000100371951**	Romidepsin active form	C/C=C1\NC(=O)[C@@H](CS)NC(=O)[C@@H](C(C)C)CC(=O)C[C@@H](/C=C/CCS)OC(=O)[C@H](C(C)C)NC1=O
**ZINC000001543873**	Vorinostat	O=C(CCCCCCC(=O)Nc1ccccc1)NO
**ZINC000011616925**	Pralatrexate stereoisomer (CIP: 4R; 25S)	C#CC[C@H](Cc1cnc2nc(N)nc(N)c2n1)c1ccc(C(=O)N[C@@H](CCC(=O)O)C(=O)O)cc1
**ZINC000019632618**	Imatinib	Cc1ccc(NC(=O)c2ccc(CN3CCN(C)CC3)cc2)cc1Nc1nccc(-c2cccnc2)n1
**ZINC000001996784**	Mirabegron	Nc1nc(CC(=O)Nc2ccc(CCNC[C@H](O)c3ccccc3)cc2)cs1
**ZINC000043200832**	Edoxaban	CN1CCc2nc(C(=O)N[C@@H]3C[C@@H](C(=O)N(C)C)CC[C@@H]3NC(=O)C(=O)Nc3ccc(Cl)cn3)sc2C1
**ZINC000001529323**	Methotrexate	CN(Cc1cnc2nc(N)nc(N)c2n1)c1ccc(C(=O)N[C@@H](CCC(=O)O)C(=O)O)cc1
**ZINC000003986735**	Dasatinib	Cc1nc(Nc2ncc(C(=O)Nc3c(C)cccc3Cl)s2)cc(N2CCN(CCO)CC2)n1
**ZINC000030691754**	Betrixaban	COc1ccc(NC(=O)c2ccc(C(=N)N(C)C)cc2)c(C(=O)Nc2ccc(Cl)cn2)c1
**ZINC000003916214**	Neratinib	CCOc1cc2ncc(C#N)c(Nc3ccc(OCc4ccccn4)c(Cl)c3)c2cc1NC(=O)/C=C/CN(C)C
**ZINC000001540998**	Pemetrexed	Nc1nc2[nH]cc(CCc3ccc(C(=O)N[C@@H](CCC(=O)O)C(=O)O)cc3)c2c(=O)[nH]1
**ZINC000003951740**	Lopinavir	Cc1cccc(C)c1OCC(=O)N[C@@H](Cc1ccccc1)[C@@H](O)C[C@H](Cc1ccccc1)NC(=O)[C@H](C(C)C)N1CCCNC1=O
**ZINC000000016154**	Ezogabine / Retigabine	CCOC(=O)Nc1ccc(NCc2ccc(F)cc2)cc1N
**ZINC000002005305**	5-Methyltetrahydrofolate	CN1c2c(nc(N)[nH]c2=O)NC[C@@H]1CNc1ccc(C(=O)N[C@@H](CCC(=O)O)C(=O)O)cc1
**ZINC000100014909**	Nintedanib	COC(=O)c1ccc2c(c1)NC(=O)/C2=C(\Nc1ccc(N(C)C(=O)CN2CCN(C)CC2)cc1)c1ccccc1
